# Loss of *MEF2C* function by enhancer mutation leads to neuronal mitochondria dysfunction and motor deficits in mice

**DOI:** 10.1186/s13024-024-00792-y

**Published:** 2025-02-07

**Authors:** Ali Yousefian-Jazi, Suhyun Kim, Jiyeon Chu, Seung-Hye Choi, Phuong Thi Thanh Nguyen, Uiyeol Park, Min-gyeong Kim, Hongik Hwang, Kyungeun Lee, Yeyun Kim, Seung Jae Hyeon, Hyewhon Rhim, Hannah L. Ryu, Grewo Lim, Thor D. Stein, Kayeong Lim, Hoon Ryu, Junghee Lee

**Affiliations:** 1 Laboratory for Brain Gene Regulation and Epigenetics, Brain Science Institute, Korea Institute of Science and Technology (KIST), Seoul, 02792 Republic of Korea; 2 Department of Integrated Biomedical and Life Science, College of Health Science, Korea University, Seoul, 02841 Republic of Korea; 3 KIST School, Division of Bio-Medical Science & Technology, University of Science and Technology (UST), Seoul, 02792 Republic of Korea; 4 Department of Biochemistry & Molecular Biology, College of Medicine, Hanyang University, Seoul, 04763 Republic of Korea; 5 Brain Science Institute, Korea Institute of Science and Technology (KIST), Seoul, 02792 Republic of Korea; 6 Department of Life Science, University of Seoul, Seoul, 02504 Republic of Korea; 7 Advanced Analysis Data Center, Korea Institute of Science and Technology (KIST), Seoul, 02792 Republic of Korea; 8 Boston University Alzheimer’s Disease Research Center and Department of Neurology, Boston University Chobanian & Avedisian School of Medicine, Boston, MA 02118 USA; 9 VA Boston Healthcare System, Boston, MA 02130 USA; 10 KHU-KIST Department of Converging Science and Technology, Kyung Hee University, Seoul, 02447 Republic of Korea; 11 Severance Biomedical Science Institute, Graduate School of Medical Science, Yonsei University College of Medicine, Seoul, 03722 Republic of Korea

**Keywords:** MEF2C, Single nucleotide polymorphism (SNP), Mitochondria, Motor neuron, Epigenetics

## Abstract

**Background:**

Amyotrophic lateral sclerosis (ALS) is a fatal neurodegenerative disorder characterized by the loss of both upper and lower motor neurons, leading to progressive paralysis. Both genetic alterations and epigenetic modifications contribute to neuronal dysfunction in the pathogenesis of ALS. However, the mechanism behind genetic mutations in the non-coding region of genes that affect epigenetic modifications remains unclear.

**Methods:**

Convolutional neural network was used to identify an ALS-associated SNP located in the intronic region of *MEF2C* (rs304152), residing in a putative enhancer element. To examine the alteration of *MEF2C* transcription by the SNP, we generated HEK293T cells carrying the major or minor allele by CRISPR-Cas9. To verify the role of *MEF2C*-knockdown (*MEF2C*-KD) in mice, we developed AAV expressing shRNA for *MEF2C* based on AAV-U6 promoter vector. Neuropathological alterations of *MEF2C*-KD mice with mitochondrial dysfunction and motor neuronal damage were observed by confocal microscopy and transmission electron microscope (TEM). Behavioral changes of mice were examined through longitudinal study by tail suspension, inverted grid test and automated gait analysis.

**Results:**

Here, we show that enhancer mutation of *MEF2C* reduces own gene expression and consequently impairs mitochondrial function in motor neurons. MEF2C localizes and binds to the mitochondria DNA, and directly modulates mitochondria-encoded gene expression. CRISPR/Cas-9-induced mutation of the *MEF2C* enhancer decreases expression of mitochondria-encoded genes. Moreover, *MEF2C* mutant cells show reduction of mitochondrial membrane potential, ATP level but elevation of oxidative stress. *MEF2C* deficiency in the upper and lower motor neurons of mice impairs mitochondria-encoded genes, and leads to mitochondrial metabolic disruption and progressive motor behavioral deficits.

**Conclusions:**

Together, *MEF2C* dysregulation by the enhancer mutation leads to mitochondrial dysfunction and oxidative stress, which are prevalent features in motor neuronal damage and ALS pathogenesis. This genetic and epigenetic crosstalk mechanism provides insights for advancing our understanding of motor neuron disease and developing effective treatments.

**Supplementary Information:**

The online version contains supplementary material available at 10.1186/s13024-024-00792-y.

## Introduction

Amyotrophic lateral sclerosis (ALS), also known as Lou Gehrig’s disease, is a progressive neurodegenerative disease characterized by the loss of motor neurons in both the upper and lower spinal cord. The loss of motor neurons leads to muscle wasting and paralysis. While around 90–95% of ALS cases are sporadic, the remaining 5–10% of cases are familial. Among familial ALS cases with a family history, roughly 20% are caused by mutations in the SOD1 gene, the first gene discovered to be involved in ALS. Additional genes linked to familial ALS include TARDBP and FUS, each accounting for 4–5% of cases. Notably, mutations in C9ORF72 contribute to nearly 40% of familial cases, while mutations in other identified and unidentified genes account for the remainder [1, 2]. Studies suggest that epigenetic factors play a role in the etiology of ALS, as well as other neurodegenerative and neuropsychiatric diseases [3–8]. Epigenetic modifications can influence various cellular processes, including mitochondrial function, energy metabolism, oxidative stress, and programmed cell death, by regulating gene expression [9]. However, the precise mechanisms by which genetic mutations in the non-coding regions of genes can trigger epigenetic modifications and contribute to the neuropathology of ALS remain incompletely understood.

Genome-wide association study (GWAS) has become a solid method for identifying intronic and intergenic genetic variations linked to various complex diseases. For ALS, GWAS has successfully pointed to genes like *C9ORF72*, *SARM*, *UNC13A*,* SCFD1*, *TBK1*, *MOBP*, *C21ORF2*, and *KIF5A* as potential risk factors [10–12]. However, although GWAS has revealed many promising leads, it can be challenging to pinpoint the exact single-nucleotide polymorphisms (SNPs) responsible for the disease, especially when they are rare, and their effects are subtle [13]. This limitation has spurred the development of post-GWAS analysis method, such as those using convolutional neural networks (CNNs) trained on epigenetic data [14]. These new approaches aim to identify functional, yet rare, risk-variants hidden within non-coding regions of the genome. One such improved CNN model, constructed with uncertain class labels, has been applied to the GWAS meta-analysis from 14,791 ALS cases and 26,898 healthy controls. This analysis successfully identified two potential risk-SNPs on chromosomes 3 and 17, opening the door for further investigation [10, 15]. Building on the largest ALS GWAS ever conducted, which encompasses data from over 20,000 patients and 59,000 healthy individuals, we scrutinized deeper into understanding the genetic roots of this devastating disease.

MEF2C is a member of the myocyte enhancer factor 2 (MEF2) family of transcription factors, primarily localized to the nucleus, and is essential for regulating gene expression in various tissues, including the brain [16]. MEF2C regulates the expression of genes critical for synaptic function and brain plasticity, making its disruption highly consequential for cognitive resilience [17]. MEF2C is involved in the development of various neuropsychiatric disorders, such as autism spectrum disorder (ASD), schizophrenia (SCZ), and Alzheimer’s disease (AD) [18]. Moreover, Mutations or deletions of MEF2C cause a rare genetic disorder, MEF2C haploinsufficiency syndrome, which is characterized by hypotonia, seizures, and cognitive impairments [19].

As reported by McLaughlin et al., the genetic correlation between ALS and SCZ was estimated to be 14.3% using GWAS data and linkage disequilibrium (LD) score regression [20]. In this study, we aimed to identify risk-SNPs associated with ALS based on genetic correlations between ALS and SCZ using a CNN model trained on separate GWAS datasets. This combined approach led us to identify four promising risk-SNPs, all hidden within the non-coding region of a gene called *MEF2C*. Intrigued by these findings, we explored whether these SNPs affect MEF2C’s activity. Interestingly, we discovered that MEF2C localized to the mitochondria of motor neurons and modulates mitochondrial DNA transcription and mitochondrial function. Lastly, we verified how a loss of *MEF2C* function contributes to neurodegeneration and motor behavior deficit in mice.

## Methods

### Convolutional neural network training

We used genetic associations from large-scale GWAS meta-analyses for ALS and SCZ. After filtering out SNPs with p-value > 5 × 10^–4^, each association block was identified on the lead-SNP with the lowest p-values and located 1 Mb apart from each other, including the 30 most significant neighboring SNPs [14]. Subsequently, epigenetic features including DHS mapping, histone modifications, target gene functions, and transcription factor binding sites (TFBS), were annotated to each SNP in 450 and 1,044 association blocks constructed for ALS and SCZ, respectively [14]. Finally, we trained a CNN model with uncertain labeling on the epigenetic features map of the association blocks [15]. The input data, comprising all association blocks (labeled 1), and control blocks constructed by shuffling the regulatory features of SNPs in the association blocks (labeled 0), were divided into training, validation, and testing sets by chromosome [15, 21]. Subsequently, the CNN model was trained with uncertain labels on the extracted epigenetic feature map using a large number of hyperparameters and an autoencoder for pre-training, as explained in the reference [15].

### Human post-mortem brain samples

Neuropathological processing of normal and ALS postmortem brain samples was performed using procedures previously established by the Boston University Alzheimer’s Disease Center (BUADC, USA). Cortical and spinal cord histopathology was verified and graded according to the criteria as described previously (Myers et al., 1988; Vonsattel et al., 1985). This study was approved for exemption by the Institutional Review Board of the BU School of Medicine (Protocol H-28974) as it included post-mortem tissues that were not classified as human subjects. The study was undergone in accordance with institutional regulatory guidelines and the principles of human subject protection outlined in the Declaration of Helsinki. Information about the brain tissues is provided in Supplementary Table 2.

### Luciferase reporter assay

Duplex of the *MEF2C* enhancer sequence containing a mutation site [5′- ATGTATTTTTCTGCAATAAGT-3′ (×2)] in human genomic DNA were synthesized (Invitrogen). Additionally, 300-bp and 500-bp fragments of human *MEF2C* promoter (from − 300 to − 500 to − 1-bp relative to the translation start site) were generated by PCR from human genomic DNA with primers shown in (Supplementary Table 3). The amplified PCR products were subcloned into the TA vector using TOPclonerTM TA core kit (Emzynomics, Korea) and confirmed by sequencing (Macrogen, Korea). The cloned sequences were released by restriction digestion with BglII and HindIII and subcloned into the pGL4.14-Enhancer-Luc vector (Promega, USA) at the identical sites.

### Enhancer mutation by CRISPR-Cas9

HEK293T cells (ATCC, CRL-11268) were cultured in Dulbecco’s Modified Eagle Medium (Welgene, USA), supplemented with 10% fetal bovine serum (Welgene, USA) and 1% antibiotic-antimycotic solution (Welgene, USA). For transfection, HEK293T cells (Invitrogen, USA) were seeded at 7.5 × 10^4^ cells per well in 48-well plates. After ~ 24 h, cells were transfected at 70–80% confluency with plasmids encoding Cas9-NG (750 ng) and sgRNA (250 ng) using Lipofectamine 2000 (1.5 µL, Invitrogen, USA). After 96 h of treatment, cells were subjected to a second treatment to increase the editing efficiencies, following the above process. At 96 h post-double treatment, we isolated single-cell-derived clonal populations using limiting dilutions. CRISPR-treated cells were seeded at 0.5 cells per well of a 96-well plate. Each cell was grown for ~ 10 days and maintained as a clonal cell line. Some cells from clonal populations were harvested using 50 µL of cell lysis buffer (50 mM Tris-HCl, pH 8.0 (Sigma-Aldrich), 1 mM EDTA (Sigma-Aldrich, USA), 0.005% sodium dodecyl sulfate (Sigma-Aldrich, USA), supplemented with 2.5 µL of Proteinase K (Qiagen, USA). Cells were lysed by incubation at 55 °C for 1 h, followed by incubation at 95 °C for 10 min. Allele frequencies of each clonal cell were measured using targeted deep sequencing. To generate NGS libraries, we performed nested PCR using KAPA HiFi HotStart DNA polymerase (Roche, USA) to label each fragment with Illumina adapters and index sequences. Final products were purified using a PCR purification kit (Enzynomics, Korea) and sequenced using a MiniSeq sequencer (Illumina, USA). NGS data were analyzed using the program as previously described [22]. The PCR primer sequences are shown in Supplementary Table 4.

### Measurement of oxygen consumption rate

The oxygen consumption rate was measured by a Seahorse XFe24 analyzer (Seahorse Bioscience, USA). A total of 4 × 10^4^ differentiated NSC-34 cells were plated in XFe24 cell culture microplates and cultured. The cartridge plate was incubated with XF Calibrant buffer for one day (37 °C, CO^2^-free); analytical medium (XF basal medium supplemented with 1mM pyruvate, 4mM glutamine, and 25mM glucose) was prepared right before analysis.

## Results

### Identification and biological characterization of ALS risk-SNPs

**Fig. 1:**
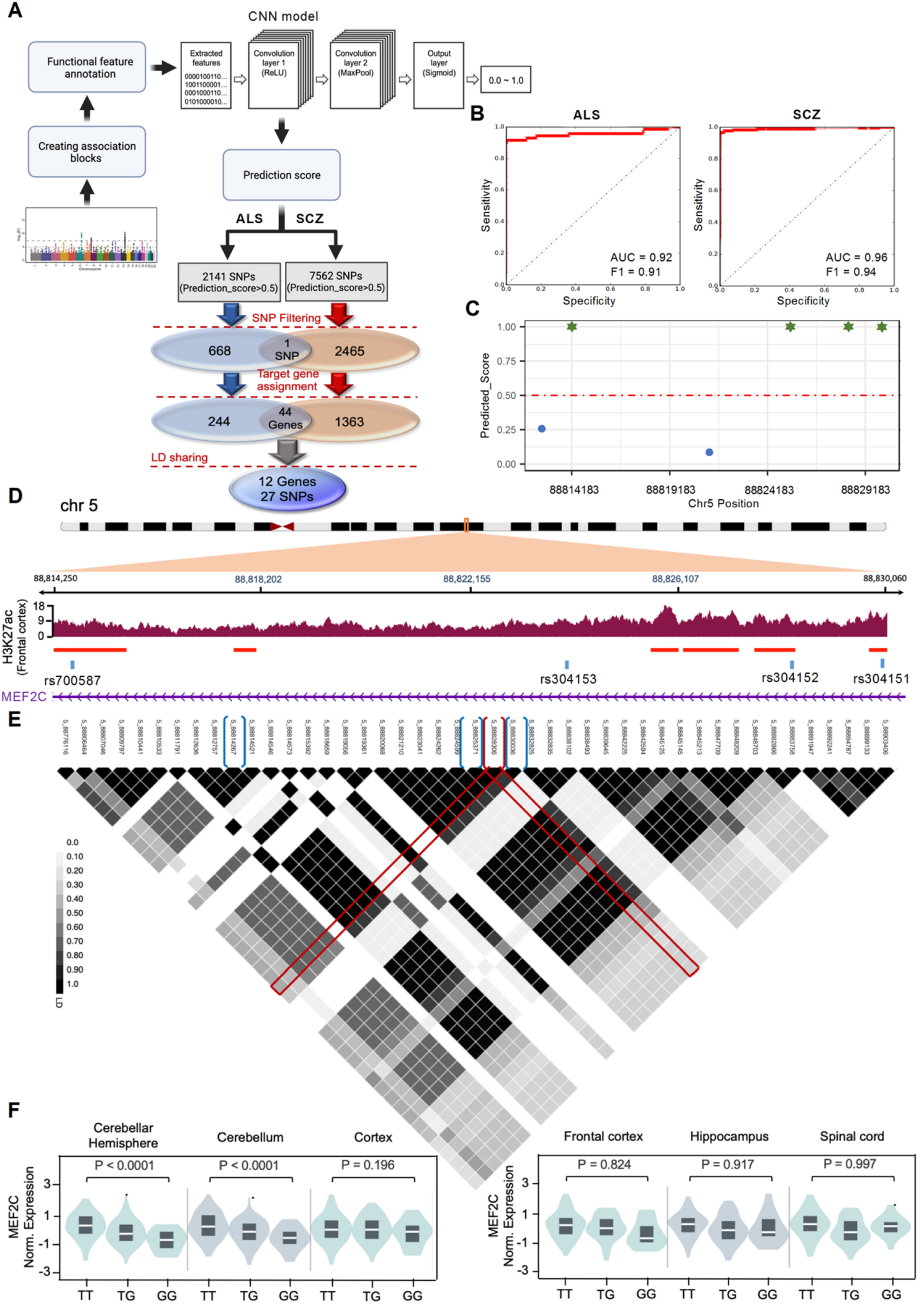
Prioritizing rs304152 as a potential functional SNP in intronic region of *MEF2C* associated with ALS. **(A)** A research flow starting from ALS and SCZ GWAS meta-analysis data and extracting functional features for each SNP, then using CNN model to initialize the candidate risk-SNPs followed by filtering, target gene assignment and LD sharing steps. **(B)** The CNN model performance for prediction of testing blocks for ALS and SCZ model. **(C)** The prediction score for each SNP in the association block contains *MEF2C* gene body. Green stars are candidate SNPs. **(D)** Active regulatory regions coverage and peaks marked by H3K27ac in frontal cortex extracted from GTEX IGV browser. **(E)** The LD plots for the region from 88.7 to 88.9 Mb in chromosome 5 including candidate SNPs. LD block structure was estimated with the Haploview software. The red brocket, rs304152, is the chosen SNP which has the high LD scores with other blue brocket candidate risk-SNPs, rs700587, rs304153 and rs304151. **(F)** Violin plots of the *MEF2C* normalized expression level according to alleles of rs304152 in different brain regions. The information was extracted from GTEX database

To identify the functional risk-SNPs using a CNN model based on the genetic correlation between ALS and SCZ, we used genetic association data from the large-scale GWAS meta-analyses for ALS and SCZ (Fig. 1A). The ALS GWAS data includes 10,031,630 imputed variants from 20,806 patients diagnosed with ALS and 59,804 control subjects [12], and the SCZ GWAS data is identified on 36,989 cases and 113,075 controls [23]. We trained the CNN model with uncertain labels on the extracted epigenetic feature map of SNPs in association and control blocks (Methods) for ALS and SCZ separately. The model’s performance was shown by the area under the curve on the test blocks for ALS and SCZ equal to 0.92 and 0.96, respectively (Fig. 1B). The CNN model predicts a prediction_score between 0 and 1 for each SNP, and those with prediction_score > 0.5 considered as the initial candidates of risk-SNPs in each block (Fig. 1C). Among 2141 and 7562 SNPs with prediction_score > 0.5 in ALS and SCZ, respectively, 669 and 2466 SNPs had been selected based on the highest prediction score in each block. Then, the target genes were assigned to each SNP using the gene body, the 3*kb* upstream of the transcription start site as a promoter region and LASSO transcriptional enhancers in brain cell lines as an enhancer region [24]. We identified 44 genes shared between ALS and SCZ (Fig. 1A). We then filtered the ALS risk-SNPs that shared linkage disequilibrium (LD) with SCZ risk-SNPs linked to these 44 genes [20]. Finally, we arrived at a list of 27 SNPs and 12 linked gene candidates associated with ALS (Supplementary Table 1). Among the 12 genes, *MEF2C* was reduced by more than 50% in frontal cortex mRNA transcriptome data from 22-month-old ALS-FUS male mice [25] (Supplementary Fig. 1). Furthermore, decreased MEF2C immunoreactivity was observed by immunofluorescence staining in cortical pyramidal neurons of ALS patients and ALS mice model (SOD1-G93A) (Supplementary Fig. 2A, C). Similarly, MEF2C immunoreactivity level significantly decreased in lower motor neurons in the lumbar spinal cord of both ALS patients and the SOD1-G93A mouse model (Supplementary Fig. 2B, D). Four candidate risk-SNPs, rs700587, rs304151, rs304152 and rs304153, located in the intronic region of the *MEF2C* gene, were identified as the risk-SNPs by our model. The rs304152 located in H3K27ac peak region which is labeled as an active enhancer marker [26], and is in the strong LD with other three SNPs, as well (Fig. 1D-E). The Hi-C data across human prefrontal cortex indicates the potential long-range chromatin interactions between rs304152 and *MEF2C* promoter region (Supplementary Fig. 3) [27]. Moreover, Genotype-Tissue Expression (GTEx) database (https://gtexportal.org) illustrates the lower *MEF2C* expression level for the risk-allele G of rs304152 in the most brain regions (Fig. 1F). Therefore, rs304152 and *MEF2C* was chosen as the ALS-associated SNP and gene for the further analysis in this study.

### Mutation in the enhancer of *MEF2C*, rs304152, reduces *MEF2C* mRNA transcription by impairing ATF4 transcription factor binding

**Fig. 2:**
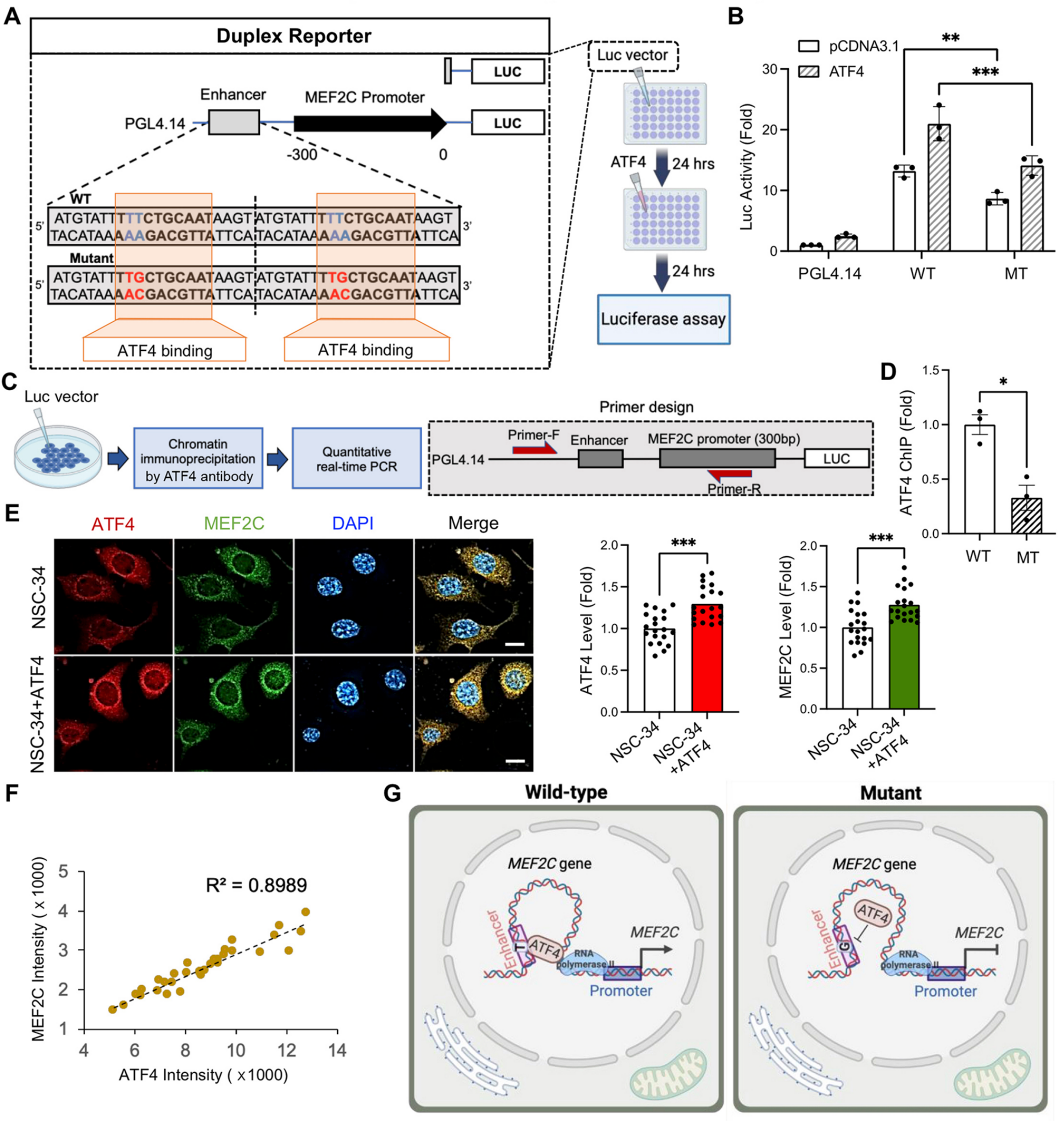
Risk-SNP (rs304152) in the *MEF2C* gene significantly reduces the transcription of its own gene by inhibiting ATF4 binding to the enhancer region. **(A)** Schematic representation of constructs used in the luciferase reporter assays. **(B)** The construct containing the rs304152-G allele (MT) showed approximately 1.7-fold less luciferase activity than the construct containing the rs304152-T allele (WT). The experiment was repeated three times. Statistics were calculated using Two-way ANOVA (*n* = 4 wells/group: **, P = 0.006; ***, P < 0.001). Error bars represent means ± SEM. **(C)** A scheme illustrating ChIP-qPCR assay for determining allele-specific DNA–protein interactions of WT and MT form of *MEF2C* enhancer region with anti-ATF4 antibody in NSC-34 cells. **(D)** ChIP-qPCR results showed less ATF4 occupancy at the MT form of *MEF2C* enhancer. Statistics were calculated using Student’s t-test: *, P = 0.01. **(E)** Immunofluorescence staining for ATF4 (red) and MEF2C (green) in NSC-34 cells transfected with ATF4 for 12 h. Right: quantification of ATF4 and MEF2C immunoreactivity levels. A total of 21 cells/group were counted (7 cells/well) from n = 3 wells/group (NSC-34 and NSC-34 + ATF4). Statistics were calculated using linear mixed model (LMM) (***, P < 0.001). **(F)** The scatter plot showed a correlation between ATF4 and MEF2C immunoreactivity levels in the cells. **(G)** A scheme summarizing that the mutant form of *MEF2C* enhancer resulted in *MEF2C* transcriptional impairment by inhibiting ATF4 transcription factor binding to the enhancer region. Scheme created with BioRender.com

Since the mutation site of rs304152 resides in a potential enhancer region, we hypothesized that this mutation may affect the promoter activity of its own gene. In this context, we generated plasmid constructs in which a duplex of a 21-bp intron sequence carrying the major or minor allele residue in the SNP-corresponding position was coupled with the human MEF2C promoter (-300 bp and − 500 bp) and a downstream luciferase reporter. Given the fact that the mutation site of the *MEF2C* enhancer harbors an ATF4 binding element, the cells were transfected with ATF4 for 24 h, and then the *MEF2C* promoter activity was measured using the Dual-Luciferase reporter assay system (Promega) (Fig. 2A). The rs304152-G allele (MT) showed less luciferase activity than the rs304152-T allele (WT) on the *MEF2C* promoter in NSC-34 cells (Fig. 2B, Supplementary Fig. 4A). The cells transfected by ATF4 revealed higher luciferase activity for both WT and MT, indicated the activator role of ATF4 binding to the region residing rs304152. ATF4 is a key ER stress transcription factor involved in the unfolded protein response (UPR) pathway [28]. Luciferase activity was elevated for the rs304152-T (WT) allele when induced by Tunicamycin, a UPR activator, whereas it remained unchanged for the rs304152-G (MT) allele (Supplementary Fig. 4B). To confirm the ATF4 occupancy in the *MEF2C* enhancer, we performed ChIP assay using the ChIP grade anti-ATF4 monoclonal antibody followed by qPCR with primers targeting the enhancer region (Fig. 2C). ChIP-qPCR results demonstrated the less occupancy of ATF4 in the MT form of *MEF2C* enhancer region (Fig. 2D). Additionally, DNA sequencing of ATF4-ChIP product confirmed that ATF4 preferentially binds to the rs304152-T allele compared to the rs304152-G allele in the MEF2C enhancer (Supplementary Fig. 4C). For further clarification on the activator role of ATF4 for *MEF2C* transcription, enforced overexpression of ATF4 was carried out by transfection of ATF4 plasmid in NSC-34 cells for 24 h. Using immunofluorescence, we confirmed that the MEF2C immunoreactivity was elevated in the cytosol compartment by increasing ATF4 immunoreactivity level (Fig. 2E). The scatter plot illustrated a high positive correlation (R^2^ = 0.89) between ATF4 and MEF2C immunoreactivity (Fig. 2F). Overall, these findings demonstrate ATF4 transcription factor preferentially bind to the WT form of *MEF2C* enhancer and promotes *MEF2C* transcription (Fig. 2G).

### Enhancer mutation, rs304152, impaired *MEF2C* transcription and mitochondrial function

**Fig. 3:**
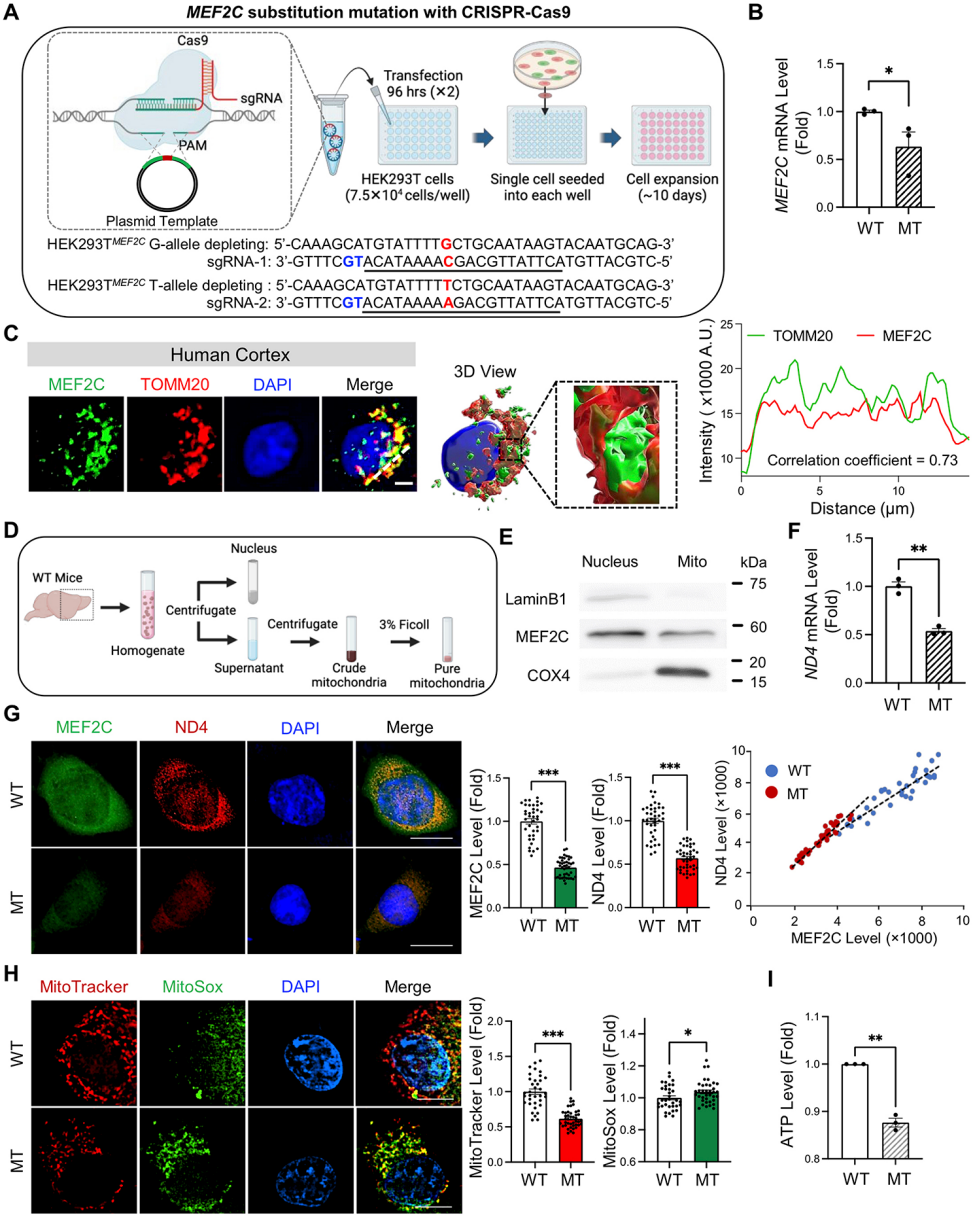
*MEF2C* enhancer mutation impairs MEF2C transcription and leads to mitochondrial dysfunction. **(A)** Demonstration of CRISPR-editing of rs304152-G mutation in HEK293T cells. Underlined sequence and blue sequence determine sgRNA and PAM, respectively. Scheme created with BioRender.com. **(B)** MEF2C mRNA level decreased in rs304152-G (MT) cells compared to rs304152-T (WT) cells. Statistics were calculated using Student’s t-test: *, P = 0.047. **(C)** Immunofluorescence staining of MEF2C (green) and outer mitochondria membrane marker, TOMM20 (red), in human cortical pyramidal neuron along with deconvolved and 3D reconstruction image made by Imaris 9 (Bitplane). Right panel shows line measurement analysis for MEF2C and TOMM20 colocalization signals. White dotted line indicates the colocalization analysis line. **(D)** Ultrafractionation of cellular compartments of mouse brain tissues. **(E)** Western blots of cell fractionation confirmed the presence of MEF2C in mitochondria in pure mitochondrial fractions. **(F)** qPCR results showed decrease of *ND4* mRNA levels in MT cells. Statistics were calculated using Student’s t-test: **, P < 0.002. **(G)** Immunofluorescence staining of MEF2C and ND4 in WT and MT cells. Scale bars (white): 5 μm. Right: densitometry analysis showed decrease of MEF2C and ND4 levels in MT cells. Scatter plot represents positive correlation between MEF2C and ND4 levels. A total of 39 cells/group were counted (13 cells/well) from *n* = 3 wells/group (WT and MT). Statistics were calculated using LMM (***, P < 0.001). **(H)** Immunostaining of MitoTracker (red) and MitoSox (green) in WT and MT cells. The nuclei were counterstained with DAPI (blue). Right: quantification of MitoTracker and MitoSox levels. A total of 36 cells/group were counted (12 cells/well) from *n* = 3 wells/group (WT and MT). Statistics were calculated using LMM (*, P = 0.037; ***, P < 0.001). **(I)** Decrease of ATP level in MT cells compared to WT cells. The experiment was repeated three times. Statistics were calculated using Student’s t-test (**, P = 0.006). Error bars represent means ± SEM

To investigate the functional consequences of rs304152, we generated HEK293T cells carrying the major or minor allele by CRISPR-Cas9 (Fig. 3A). Consistent with our hypothesis, rs304152-G (MT) cells displayed significantly lower *MEF2C* mRNA levels compared to rs304152-T (WT) cells (Fig. 3B). Moreover, the GTEX data indicated that the rs304152-G allele reduces the expression level of MEF2C target genes in the frontal cortex, which are downregulated in *Fus* knock-in mice [25, 29] (Supplementary Fig. 5). After verifying that the rs304152 mutation decreases *MEF2C* expression level, we investigated which subcellular organelle MEF2C localized in the cytosol to elucidate the specific role of MEF2C in neuron. By observing the immunofluorescence co-staining of MEF2C and TOMM20, an outer mitochondrial membrane marker, we found MEF2C localized to mitochondria in postmortem human cortex pyramidal neurons. The 3D reconstruction and line measurement results confirmed the localization of MEF2C in mitochondria (Fig. 3C). Even though mouse cell line and human tissue show a different pattern of MEF2C localization in the subcellular compartments, we found that a higher expression level of MEF2C is apparently found in the mitochondrial compartment than in the nuclear compartment (Figs. 2E and 3C). According to the quantification data, 62.7% of TOMM20- and MEF2C-double positive signals are localized to the mitochondria of cortical layer V pyramidal neurons (Supplementary Fig. 6A). We further quantitated MEF2C signals in DAB-stained cells and confirmed that approximately twice as much of MEF2C immunoreactivity is found in the cytosol compartment compared to the nuclear compartment of the cortical layer V pyramidal neurons in both normal and ALS patients (Supplementary Fig. 6B). MEF2C localization in mitochondria was observed in mouse cortical pyramidal neurons as well (Supplementary Fig. 6C). Moreover, we obtained subcellular fractions from mouse brain tissue by sucrose density centrifugation (Fig. 3D). Western blot analysis confirmed that MEF2C exist in enriched mitochondrial fractions of mouse brain tissue (Fig. 3E, Supplementary Fig. 6D). The results showed the LaminB1 and COX4 immunoreactivity mostly in nuclear and mitochondrial fractions, respectively, which indicates the mitochondrial fractions without contamination with other subcellular organelles. MEF2C was less localized in mitochondria in MT cells with smaller mitochondria network size (Supplementary Fig. 7).

We further examined the effect of MEF2C enhancer mutation on mitochondrial-encoded gene transcription and mitochondrial activity. The qPCR results showed the rs304152-G allele had an impaired effect on *ND4* mRNA level (Fig. 3F). The immunofluorescence staining results confirmed the decrease of MEF2C and ND4 immunoreactivity levels in MT cells, and the positive correlation between MEF2C and ND4 immunoreactivity levels (Fig. 3G). Activation of the UPR with Tunicamycin in WT cells induced ND4 expression, while ND4 immunoreactivity remained unchanged in MT cells (Supplementary Fig. 8). Mitochondria membrane potential decreased, and mitochondria oxidative stress elevated in MT cells (Fig. 3H). Finally, we measured ATP level in cells using luminescence and verified the ATP level significantly decreased in MT cells (Fig. 3I). Moreover, we assessed mitochondrial dysfunction in human neuroblastoma SH-SY5Y cells transfected with Cas9-NG, sgRNA, and ssODN plasmids along with CMV-GFP as a transfection marker (Supplementary Fig. 9A). The immunostaining results showed a decrease in ND4 immunoreactivity in MT^SH-SY5Y^ cells (Supplementary Fig. 9B). Mitochondrial oxidative stress was also increased in MT^SH-SY5Y^ cells, as observed in HEK293T cells (Supplementary Fig. 9C). Altogether, rs304152-G reduced *MEF2C* expression, resulting mitochondria genes dysregulation and mitochondrial dysfunction.

### MEF2C targets mitochondria genome and regulates mitochondria function

**Fig. 4:**
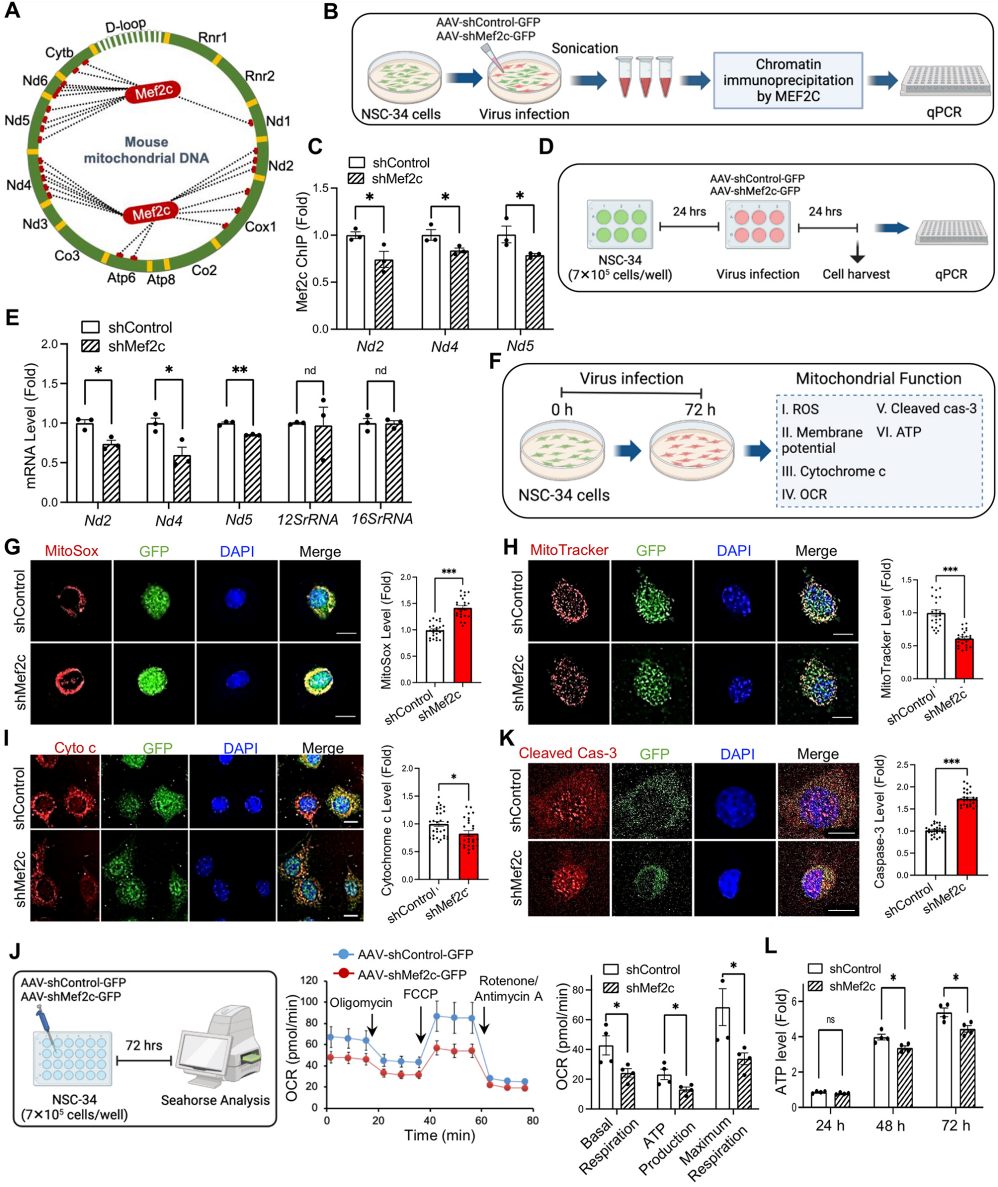
MEF2C regulates mitochondria gene expression and mitochondrial function in motor neuron cell line (NSC-34). **(A)** MEF2C transcription factor binding sites at mouse mitochondrial DNA detected by TRANSFAC 6.0-based algorithm, Patch 1.0. **(B)** A scheme illustrating performing MEF2C ChIP assay on NSC-34 cells infected by *Mef2c*-KD (shMef2c) and control (shControl) viruses, and measuring level of specific DNA in ChIP samples in mitochondria genes by qPCR. **(C)** MEF2C ChIP-qPCR showed Mef2c occupancy level in *Nd2*, *Nd4* and *Nd5* mitochondrial genes in shControl and shMef2c cells. Data generated from 3 samples which are duplicated and normalized to input DNA. Statistics were calculated using Student’s t-test (ND2, *, P = 0.04;  ND4, *, P = 0.047; ND5, *, P = 0.05). **(D)** A scheme illustrating performing qPCR on NSC-34 cells infected by shControl and shMef2c viruses and harvested after 24 h. **(E)** qPCR results showed mRNA level of mitochondria genes, *Nd2*, *Nd4*, *Nd5*, decreased by *Mef2c*-KD. Statistics were calculated using Student’s t-test (ND2, *, P = 0.035; ND4, *, P = 0.05; *ND5,* **, P = 0.025). **(F)** A scheme illustrating a series of experiments to evaluate mitochondrial function in in NSC-34 cells infected by *Mef2c*-KD virus. Immunostaining of **(G)** MitoSox (red), **(H)** MitoTracker (red) and **(I)** cytochrome c (Cyto c) (red) in GFP^+^ NSC-34 cells infected by *Mef2c*-KD virus. The nuclei were counterstained with DAPI (blue). Scale bars (white): 5 μm. Right: quantification of MitoSox, MitoTracker and cytochrome c levels in GFP^+^ cells. A total of 30 cells/group were counted (10 cells/well) from *n* = 3 wells/group (shControl and shMef2c). Statistics were calculated using LMM (*, P = 0.012; ***, P < 0.001). **(J)** Cellular respiration rate of differentiated NSC-34 cells infected by *Mef2c*-KD virus measured by a Seahorse XFe24 analyzer. Right: quantitative analysis of the basal and maximum respiratory rate, and ATP production amount of GFP^+^ cells. Statistics were calculated using Student’s t-test (*n* = 4 wells/group: *, P < 0.05 *,*). **(K)** Immunofluorescence staining of cleaved caspase-3 in NSC-34 cells infected by *Mef2c*-KD virus. Scale bars (white): 5 μm. Right: densitometry analysis for GFP^+^ cells. A total of 30 cells/group were counted (10 cells/well) from *n* = 3 wells/group (shControl and shMef2c). Statistics were calculated using LMM (***, P < 0.001). **(L)** Decrease of ATP level by *Mef2c*-KD at 24, 48 and 72 h after the virus infection in NSC-34 cells. Statistics were calculated using Student’s t-test (*n* = 4 wells/group: *P* = 0.097 at 24 h; *, P = 0.025 at 48 h; *, P = 0.023 at 72 h). Error bars represent means ± SEM

After confirming that the *MEF2C* enhancer mutation impaired *MEF2C* and mitochondrial gene transcription, we examined the effect of reduced MEF2C function in vitro. Given that MEF2C is a known transcription factor, we investigated the existence of MEF2C binding elements in mitochondria genes. Using the TRANSFAC 6.0-based algorithm Patch 1.0, we predicted the MEF2C-binding sites in mouse mitochondrial DNA (Fig. 4A). To confirm the MEF2C occupancy level in mitochondrial genomes, we performed MEF2C ChIP-qPCR in NSC-34 cells infected by *Mef2c* knock-down (*Mef2c*-KD) virus, pAAV-hSyn(pro)-shMef2c-GFP (Fig. 4B). The *Mef2c*-deficient cells showed reduced Mef2c occupancy in *Nd2*, *Nd4* and *Nd5* genes (Fig. 4C). DNA sequencing of MEF2C-ChIP product verified *Nd2*, *Nd4* and *Nd5* mitochondrial genes (Supplementary Fig. 10A). Moreover, MEF2C occupancy in mitochondrial genomes was confirmed by previous MEF2C-ChIP sequencing data in human umbilical vein cells (GSM809016) and mouse prefrontal cortex (GSM5244364) as well (Supplementary Fig. 10B). To verify whether MEF2C binds to the DNA inside of intact mitochondria or not, we performed nuclear and mitochondria fractionation from the cortex of WT mice. Then, we ran MEF2C-ChIP and qPCR to quantitate MEF2C-DNA occupancy with primers specific to *Nd4*, a mitochondria-encoded gene, and *Rgs6*, a nuclear-encoded gene. *Rgs6* is targeted by MEF2C in cortical neurons of mice [30]. As a result, our ChIP and qPCR analysis confirmed that the MEF2C-DNA occupancy in the *Nd4* gene was only found in the mitochondria fraction while the MEF2C-DNA occupancy in the *Rgs6* gene was only detected in the nuclear fraction (Supplementary Fig. 11). *Mef2c*-KD cells exhibited decreased mRNA levels of *Nd2*, *Nd4*, and *Nd5*, while MEF2C overexpression (*MEF2C*-O/E) cells showed increased expression (Fig. 4D-E, Supplementary Fig. 12A-B). We further examined that *Mef2c*-KD significantly decreases *Nd4* mRNA levels in a shMef2c-dose dependent manner. In this context, we verified that *Mef2c*-KD, as a similar experimental model of reduced *Mef2c* expression due to SNP (rs304152), directly and negatively affects mitochondria gene transcription (Supplementary Fig. 12C). MEF2C not only directly regulates mitochondrial genes but also indirectly influences mitochondrial function by controlling the expression of nuclear-encoded genes, such as mitochondrial transcription factor A (TFAM), translocase of the outer mitochondrial membrane 20 (TOMM20), mitofusin 1 (MFN1), and dynamin-related protein 1 (DRP1) at the mRNA level (Supplementary Fig. 12D-F). TFAM binds to mitochondrial DNA (mtDNA) and involves in initiating and regulating transcription of mtDNA. TOMM20 is a receptor on the outer mitochondrial membrane involves in the import of precursor proteins into mitochondria, and MFN1 and DRP1 are involved in the balance of mitochondrial fusion and fission. In order to examine whether MEF2C functions as a monomer or dimer in the mitochondria, we crosslinked the mitochondria fraction with 1% glutaraldehyde and ran Western blot analysis. As a result, we found that both monomer and dimer of MEF2C signals were detected in the mitochondria fraction as well as in the nucleus fraction (Supplementary Fig. 6E).

To investigate the consequences of MEF2C deficiency in mitochondrial function, we performed a series of immunocytochemistry to evaluate mitochondrial ROS, membrane potential, cleaved caspase-3 and cytochrome c levels, and oxygen consumption rate seahorse measurement (Fig. 4F). The mitochondrial superoxide (MitoSox) and mitochondrial membrane potential (MitoTracker) levels were analyzed by comparing the fluorescence staining in NSC-34 cells infected by *Mef2c*-KD and *MEF2C*-O/E viruses (Fig. 4G-H and Supplementary Fig. 12G-H). *Mef2c*-KD resulted in an increase in mitochondrial ROS generation and a decrease in mitochondrial membrane potential in NSC-34 cells (Fig. 4G-H). Moreover, the immunocytochemistry results showed a decrease in cytochrome c level in *Mef2c*-KD cells (Fig. 4I). Then, considering the mitochondria as a main subcellular organelle for cellular respiration and energy production, we verified the *Mef2c* deficiency effects on cellular respiration rate by a Seahorse analyzer [31]. The results confirmed the significant decrease of basal and maximum respiration and mitochondrial ATP production rates in *Mef2c*-KD cells (Fig. 4J). In addition, by immunofluorescence labeling, we confirmed an increase in active caspase-3 immunoreactivity in *Mef2c*-KD cells, which indicates induce of apoptotic cell death by *Mef2c* deficiency (Fig. 4K). Finally, the cell viability assay verified a reduction in ATP level by 15% and 17% at 48 and 72 h after *Mef2c*-KD virus infection in NSC-34 cells, respectively, suggesting elevation of cells energy demand (Fig. 4L). Altogether, MEF2C loss of function increases the mitochondrial oxidative stress, and decrease ATP production, leading to the accumulation of cell damage and eventual cell apoptosis.

### In vivo knockdown of *Mef2c* induces mitochondria dysfunction, motor neuronal damage, and altered excitability in mice

**Fig. 5:**
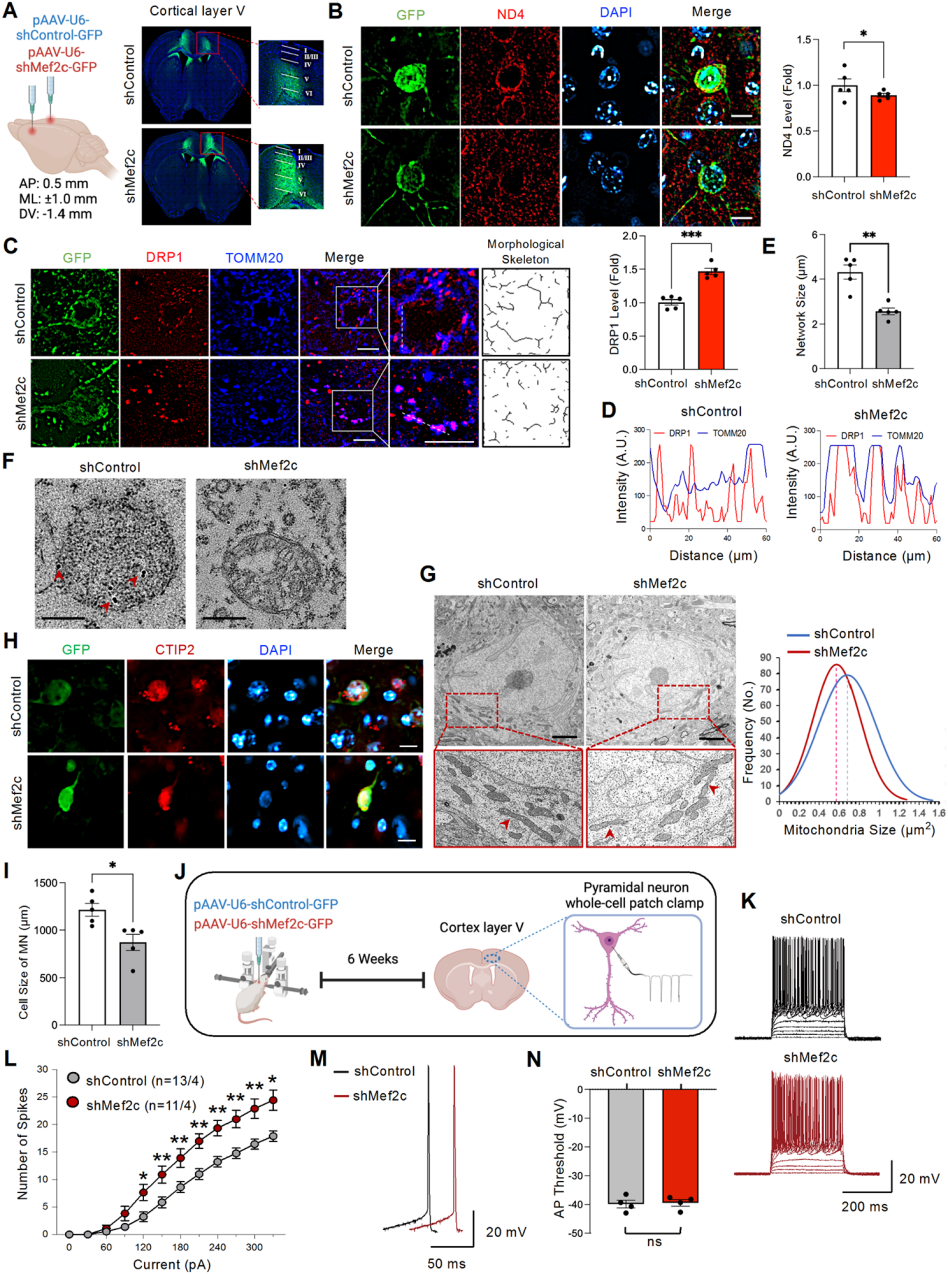
*Mef2c*-KD induces mitochondrial dysfunction and motor neuronal damage, and alters motor neuron excitability in the cortical layer V of mice. **(A)** The pAAV-hSyn(pro)-shControl-GFP (shControl) or pAAV-hSyn(pro)-shMef2c-GFP (shMef2c) viruses was delivered into cortical layer V by bilateral stereotaxic injection method. **(B)** Immunostaining of ND4 and DAPI in shControl and shMef2c mice. Scale bars (white): 5 μm. Right: densitometry analysis of ND4 immunoreactivity level in GFP^+^ cells. A total of 30 cells/group were counted (6 cells/mouse) from *N* = 5 mice/group (shControl and shMef2c). Statistics were calculated using Student’s t-test (*, P  < 0.05). **(C)** Immunostaining of DRP1 and TOMM20 in shControl and shMef2c mice. Scale bars (white): 5 μm. Right: skeletonized images of mitochondria morphological structure using TOMM20 signals, and densitometry analysis of DRP1 immunoreactivity level in mitochondria of GFP^+^ cells. A total of 30 cells/group were counted(6 cells/mouse) from *N* = 5 mice/group (shControl and shMef2c). Statistics were calculated using Student’s t-test (***, P < 0.001). **(D)** Line measurement analysis for DRP1 and TOMM20 colocalization signals in shControl and shMef2c-transduced cortical pyramidal neuron. **(E)** Results of mitochondria network size analysis performed by MiNA plugin in Fiji ImageJ software. A total of 10 cells/group were counted (2 cells/mouse) from *N* = 5 mice/group (shControl and shMef2c). Statistics were calculated using Student’s t-test (**, P = 0.003). **(F)** Representative TEM images showed the presence of immunogold particles (red head arrows) in mitochondria of the pyramidal neurons layer V in shControl mice. Scale bars: 200 nm. **(G)** Representative TEM images showed mitochondria fission in shMef2c group. Scale bars: 2 μm. Right: a normalized curve for quantitative analysis of mitochondria size in the cortical pyramidal neurons of shControl and shMef2c mice. A total of *n* = 200 mitochondria/20 neurons/group were counted from *N* = 5 mice/group (shControl and shMef2c). **(H)** Immunostaining of CTIP2 in shControl and shMef2c mice. Scale bars: 5 μm. **(I)** Quantification analysis on the cell size of cortical layer V pyramidal neurons in GFP^+^ cells. A total of 15 cells/group were counted (3 cells/mouse) from *N* = 5 mice/group (shControl and shMef2c). Statistics were calculated using Student’s t-test (*, P  < 0.05). **(J)** A scheme illustrating performing whole-cell patch clamp on cortical layer V pyramidal neurons for shControl and shMef2c mice. **(K)** Representative recordings of action potentials from motor cortex layer V pyramidal neurons induced by step current injection, ranging from 0 to 330 pA (Scale bar: 20 mV, 200 ms). **(L)** shMef2c increased the number of action potentials triggered by a series of current injection (30-pA increment, 12 steps). shControl, *n* = 13 neurons/4 mice; shMef2c, *n* = 11 neurons/4 mice). Statistics were calculated using Student’s t-test (60(pA), *P* = 0.438; 90(pA), *P* = 0.088; 120(pA), *, P = 0.013; 150(pA), **, P = 0.009; 180(pA), **, P = 0.01; 210(pA), **, P = 0.001; 240(pA), **, P = 0.001; 270(pA), **, P = 0.002; 300(pA), **, P = 0.002; 330(pA), *, P = 0.03). **(M)** Representative traces of individual action potential (Scale bar: 20 mV, 50 ms). **(N)** The threshold to initiate action potentials is not altered in shMef2c group (shControl, *n* = 13 neurons/4 mice; shMef2c, *n* = 11 neurons/4 mice). Statistics were calculated using Student’s t-test, *P* = 0.828. Error bars represent means ± SEM

Since the alignment of the *MEF2C* enhancer sequence among human, primates, canines, and mice indicated a lack of conservation across species, and the rs304152-G allele represents a unique human-specific phenotype (Supplementary Fig. 13), we applied an AAV-shRNA-mediated *MEF2C* knockdown system for in vivo studies. To verify whether *Mef2c* deficiency affects mitochondrial dysfunction in mice, we delivered the AAV-sh*Mef2c* virus into the cortical layer V of wild-type mice via bilateral stereotaxic injection (Fig. 5A and Supplementary Fig. 14). Immunofluorescent labeling revealed a decrease in MEF2C immunoreactivity in GFP^+^ cells within cortical layer V pyramidal neurons of shMef2c mice (Supplementary Fig. 15A). Immunostaining with anti-ND4 confirmed that *Mef2c*-KD decreased ND4 immunoreactivity level in mice (Fig. 5B). Consistent with the in vitro observation of reduced *Mfn1* and increased *Drp1* mRNA levels, suggesting a shift towards excessive mitochondrial fission following *Mef2c*-KD (Supplementary Fig. 12D), in vivo immunostaining with anti-DRP1 as the master regulator of mitochondrial fission showed the increase of DRP1 immunoreactivity level in mitochondria of cortical pyramidal neurons in shMef2c mice (Fig. 5C). Colocalization of DRP1 and the mitochondria outer membrane marker in cortical pyramidal neurons indicates execution of the mitochondria fission process in shMef2c mice [32] (Fig. 5D). The mitochondria fragmentation was quantified by measuring mitochondria network size with TOMM20 signals using MiNA plugin in Fiji ImageJ software (Fig. 5E). To confirm our confocal microscopic observations, we further investigated the MEF2C localization to mitochondria and mitochondria size using transmission electron microscopy (TEM). The immunogold labeling results showed gold-labeled particles in the mitochondria of cortical motor neurons in control mice which reduced from neuronal mitochondria of *Mef2c*-deficient mice (Fig. 5F). Control staining without the primary antibody lacked gold particles within the mitochondria (data not shown). Moreover, TEM images showed mitochondria fragmentation in cortical pyramidal neurons of shMef2c mice (Fig. 5G). As previously established, MEF2C, as a transcription factor, plays a role in regulating several nuclear-encoded genes [29]. In the current study, we identified several nuclear-encoded target genes (*Hs3st2*, *Vipr1*, *Pde1a*, *Satb2*, *Slc17a7*) that are regulated by MEF2C by analyzing public data from *Cosgrove et al.* [33]. (Supplementary Fig. 15B). *Hs3st2* is a marker for a cluster of excitatory neurons in cortical layer V that is upregulated in *Mef2c*-cKO mice and plays a role in axonal outgrowth of motor neuron [33, 34]. Our immunostaining results showed an elevation in HS3ST2 immunoreactivity in *Mef2c*-KD mice (Supplementary Fig. 15C). Finally, the immunohistochemistry with anti-CTIP2 antibody as a marker for pyramidal neurons layer V showed the shrinkage of pyramidal neurons in shMef2c mice, indicating upper motor neuron damage (Fig. 5H-I) [35]. Next, we performed intrathecal injection of *Mef2c*-KD virus in mice and examined the lower motor neuron pathology (Supplementary Fig. 16A). Immunofluorescent labeling confirmed a decrease in MEF2C immunoreactivity levels in GFP^+^ cells in shMef2c mice (Supplementary Fig. 16B). The lower motor neuron *Mef2c*-deficient mice showed a decrease in ND4 but an increase in DRP1 immunoreactivity levels (Supplementary Fig. 16C-E). TDP-43 protein mislocalization from its normal nuclear compartment to the cytoplasm, along with cytoplasmic aggregation, plays an important role in ALS pathology [36]. The *Mef2c*-KD mice showed TDP-43 mislocalization to the cytoplasm as puncta structures in the motor neurons of the lumbar spinal cord (Supplementary Fig. 16F). The marked reduction of ventral neuronal size can be seen in Nissl-stained tissue sections from the lumbar spinal cord of *Mef2c*-KD mice (Supplementary Fig. 16G). Altogether, *Mef2c* deficiency in both upper and lower motor neurons leads to the downregulation of mitochondria genes, mitochondria fragmentation and motor neuronal damage in mice.

Finally, we examined a potential change in intrinsic excitability in upper motor neurons at cortical layer V of *Mef2c*-KD mice (Fig. 5J). Electrophysiological analysis showed that the current step triggered significantly higher number of action potentials in the shMef2c group, indicating that knockdown of *Mef2c* increases the intrinsic neuronal excitability in motor cortex layer V pyramidal neurons (Fig. 5K-L). However, the threshold for firing action potentials was not altered by *Mef2c*-KD (Fig. 5M-N).

### *Mef2c*-KD exhibits motor neuron disease-like behaviors in mice

**Fig. 6:**
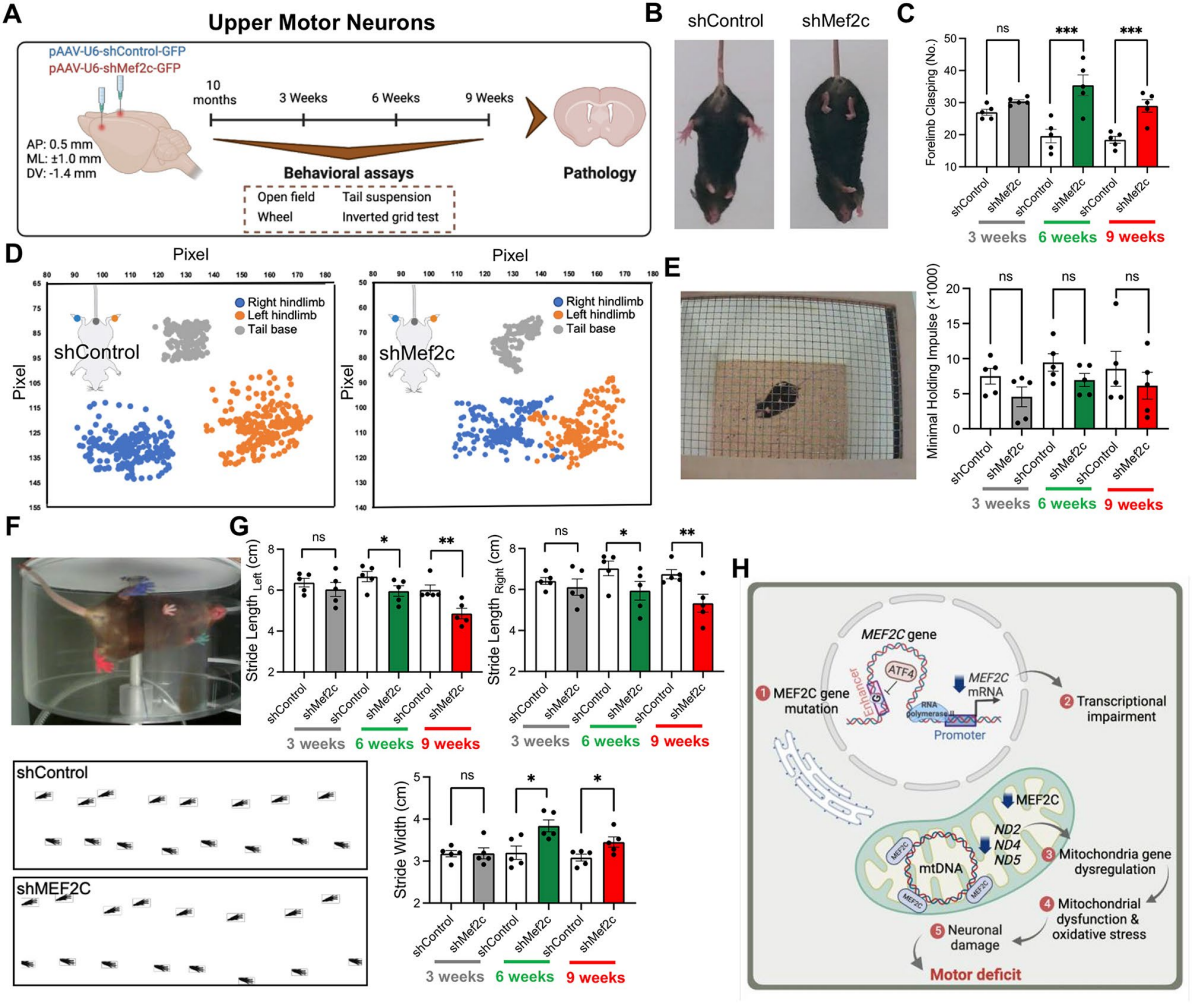
*Mef2c*-KD shows motor neuron disease-like behaviors in mice. **(A)** A scheme illustrating delivery of AAV-shRNA-Control-GFP (shControl) or AAV-shRNA-Mef2c-GFP (shMef2c) viruses into the cortical layer V of WT mice and performing longitudinal behavioral study 3, 6 and 9 weeks after injection. **(B)** Representative still images of the tail suspension test for shControl and shMef2c-transduced mice. **(C)** The number of forelimbs clasping in tail suspension test in 3-, 6- and 9-weeks post-injection. Statistics were calculated using repeated measures ANOVA (*N* = 5 mice/group for shControl and shMef2c) (3weeks, *P* = 0.51; 6 and 9weeks, ***, P < 0.001). **(D)** Aggregated coordinate plots in the first 10 s of tail suspension test for shControl and shMef2c-transduced mice. **(E)** A representative still image of the inverted grid test. Right: the minimal holding impulse. Statistics were calculated using repeated measures ANOVA (*N* = 5 mice/group for shControl and shMef2c) (3weeks, *P* = 0.387; 6weeks, *P* = 0.37; 9weeks, *P* = 0.848). **(F)**  A representative still image of the accelerated wheel test. Bottom: the computer-assisted footprint in wheel running test for a representative mouse in each group. **(G)** Gait analysis showed shorter stride length and wider stride width in shMef2c mice. Statistics were calculated using repeated measures ANOVA (*N* = 5 mice/group for shControl and shMef2c, *n* = 10 steps/mouse). Significantly different at *, *P* < 0.05; **,* P* <0.01. Error bars represent means ± SEM. **(H)** A scheme proposing how *MEF2C* enhancer SNP impairs *MEF2C* transcription, resulting in mitochondrial dysfunction and inducing oxidative stress which makes motor neuronal damage and motor deficit. Scheme created with BioRender.com

Finally, we examined whether knockdown of *Mef2c* in upper motor neurons and lower motor neurons affects the motor behavior in mice. The longitudinal behavioral study including open field, tail suspension, accelerated wheel, and inverted grid tests, was performed 3, 6 and 9 weeks after delivering *Mef2c*-KD virus into the cortical layer V of mice by bilateral stereotaxic injection method (Fig. 6A). *Mef2c*-KD mice showed abnormal hindlimb extension reflex compared to control mice (Fig. 6B and Supplementary Fig. 17A). The frequency of forelimb movements was higher in shMef2c mice at 6 and 9 weeks after injection (Fig. 6C). Hindlimb posture is visually reflected by temporally aggregated coordinate plots for the first 10 s of tail suspension (Fig. 6D). The inverted grid test was performed as a method to assess muscle strength using all four limbs. The results showed less minimal holding impulse time (body mass × cling time) for *Mef2c*-KD mice, though not statistically significant, a trend towards reduced holding time was observed (Fig. 6E). The computer-assisted footprint analysis allows us to calculate gait characteristics while the mice walk inside of an accelerating wheel (Fig. 6F and Supplementary Fig. 17B). Quantitative analysis of footprint patterns revealed motor coordination impairment in *Mef2c*-KD mice from 6 weeks post-injection, by wider stride width and shorter stride length (Fig. 6G). Open field test results showed normal behavior in locomotion and anxiety levels in shMef2c mice (Supplementary Fig. 18).

We also examined the motor behavioral changes in lower motor neurons *Mef2c*-KD mice by performing behavioral tests 3, 6 and 9 weeks after intrathecal virus injection (Supplementary Fig. 19A). The *Mef2c*-KD mice showed abnormal hindlimb clasping and increase of number of forelimb clasping (Supplementary Fig. 19B-D). The inverted grid test showed less minimal holding impulse time in *Mef2c*-KD mice (Supplementary Fig. 19E). The computer-assisted gait analysis revealed significantly decrease of stride length and an increase in stride width in *Mef2c*-KD mice hindlimbs (Supplementary Fig. 19F). Furthermore, *Mef2c*-KD mice showed fewer spontaneous movements, specifically decrease of the number of rearing, in the cylinder test at 3- and 6-weeks post-injection (Supplementary Fig. 19G). The behavior tests result at 9-weeks post-injection showed no significant differences between shControl and shMef2c mice because of render inoperative of intrathecal AAV-shRNA virus injection [37] (Supplementary Fig. 20). Open field test results showed normal behavior in locomotion and anxiety levels in lower motor neuron *Mef2c*-KD mice (Supplementary Fig. 21). Altogether, the motor behavior tests demonstrated motor neuron disease-like behaviors in both upper and lower motor neurons *Mef2c*-deficient mice.

We summarize our findings that *MEF2C* enhancer ALS-associated SNP (rs304152) impairs *MEF2C* transcription by inhibiting ATF4 transcription factor binding. Downregulation of *MEF2C* directly dysregulates mitochondria-encoded genes transcription. Finally, MEF2C deficiency leads to mitochondrial dysfunction, oxidative stress and consequently motor neuronal damage, which is the main reason for ALS pathogenesis (Fig. 6H).

## Discussion

This study identifies a novel ALS-associated intronic SNP (rs304152) located in active regulatory regions marked by H3K27ac in humans using a CNN model [38]. The rs304152 is located in the enhancer region of the *MEF2C* gene. The minor allele (T/G) of rs304152 impaired ATF4 binding to *MEF2C* enhancer region, resulting a reduction of the transcriptional activity in its own gene expression [39]. ATF4 is one of the main transcriptional effectors of the UPR, modulating cellular stress caused by protein misfolding [40]. The rs304152-G allele impairs the effect of UPR activation on *MEF2C* and *ND4* induction. The rs304152-G allele impairs the effect of UPR activation on *MEF2C* and *ND4* induction. The enhancer genetic mutation disrupts the interaction between the enhancer and transcription factor and effects promoter activity [41]. Our data indicates, for the first time, that the rs304152 SNP located in the intronic enhancer region of *MEF2C* affects its own gene expression via an epigenetic regulatory pathway of gene transcription.

MEF2C, a neuron-specific transcription factor, is known to be localized to the nucleus and plays a crucial role in the regulation of gene expression [16]. Previous studies have proposed that MEF2 is a target of mitochondrial apoptotic caspases, while also regulating mitochondrial genome transcription [42]. MEF2D has been shown to bind to a MEF2 consensus site in mtDNA, leading to the induction of *ND6* transcription [43]. Interestingly, in the current study, we discovered mitochondrial localization of MEF2C in cortical pyramidal neurons in human and mouse brains. We verified that MEF2C directly regulates mitochondrial genes and also indirectly modulates mitochondrial function by regulating nuclear-encoded genes, including *TFAM*, *TOMM20*, *DRP1*, and *Mfn1*, in vitro and in vivo. In addition to MEF2C’s role in mitochondrial function, we found that the nuclear-encoded gene *HS3ST2*, which is induced in *Mef2c*-deficient mice, plays a role in motor neuronal axon outgrowth. A previous study showed that knockdown of *Hs3st2* in a zebrafish model of tauopathy expressing hTau^P301L^ significantly reduced the level of phosphorylated tau in both the brain and the spinal cord and subsequently recovered the axonal length of motor neurons [34]. In this context, *Hs3st2* induction by the loss of *MEF2C* function may negatively affect the axonal function of motor neurons. Given that a previous study identified MEF2C-MEF2D heterodimers in HEK293 cells, our results indicate that MEF2C may function as a monomer or dimer in the mitochondria as in the nucleus [44]. It is interesting to note that the heterodimer of more than one MEF2 protein does not exhibit better transcriptional activity than the homodimer of a single MEF2 protein [45]. In this context, either monomer or dimer of MEF2C is more transcriptionally active in mitochondrial gene regulation, needs to be determined in future studies. Mitochondrial dysfunction has been implicated in various neurodegenerative diseases [46]. Previous work has shown that TFAM links the nuclear transcription response to mtDNA content and has a role in supporting mitochondrial respiratory function [47]. TOMM20 is a protein that plays a crucial role in mitochondrial respiration, ATP production, and the transport of proteins into mitochondria [48]. We determined that the knockdown of *Mef2c* decreases mitochondrial membrane potential and respiration function, leading to a loss of ATP production. In turn, *Mef2c* deficiency increased mitochondrial oxidative stress and induced neuronal apoptosis in NSC-34 motor neurons [49]. Furthermore, *Mef2c*-KD mice showed reduction of mitochondria gene expression and excessive mitochondrial fission by the translocation of Drp1 from cytosol to the mitochondrial outer membrane in both upper motor neurons (pyramidal neurons at the cortical layer V) and lower motor neurons (motor neurons at the lumbar spinal cord). Our results suggest that the loss of MEF2C function dysregulates the expression of mitochondria-encoded genes (*ND2*, *ND4* and *ND5*), alters mitochondrial morphology, and induces mitochondrial dysfunction, leading to motor neuronal damage similar to the pathology found in ALS mice [50].

A previous study by Mitchell et al. has demonstrated that MEF2C deficiency is a genetic and epigenetic risk factor for SCZ [51]. Rescue of Mef2c function improves cognitive performance in working memory and object recognition in mice [51]. On the other hand, a recent report shows that the decrease of MEF2C-mediated neuronal transcription by the activation of microglial cyclic GMP–AMP synthase and type I interferon (IFN-I) signaling declines cognitive function in AD [17]. In addition, Li et al. found a de novo autism-associated mutation in the *MEF2C* gene and constructed *Mef2c*-mutant mice showing autistic-like behaviors that can be rescued by CRISPR-Cas9 genome editing [52]. *MEF2C* deletion at 5q14.3 is the primary cause of MEF2C haploinsufficiency syndrome, which is typically associated with neurodevelopmental disorders [19]. The overlapping features of synaptic dysfunction and neuronal vulnerability to degeneration observed in both MEF2C haploinsufficiency syndrome and ALS suggest that MEF2C loss of function may contribute to ALS pathogenesis through similar pathways [53]. On the other hand, TDP-43 protein mislocalization from the nucleus to the cytoplasm and subsequent cytoplasmic aggregations is a hallmark of ALS pathology [54]. *MEF2C* loss of function leads to the mislocalization of TDP-43 protein to the cytoplasm as puncta structures in motor neurons in the lumbar spinal cord of mice. Finally, Arosio et al. measured and compared *MEF2C* mRNA levels in peripheral blood mononuclear cells (PBMCs) from patients with mutations in the *SOD1* gene [55]. Their findings indicate that MEF2C protein levels were unchanged, but MEF2C immunoreactivity was more frequently observed outside the nuclei in sporadic ALS PBMCs, while in healthy control, the signal was more diffused within the nucleus. Their data suggest that MEF2C is more abundantly localized in the cytosolic compartment, which indirectly supports our novel finding that the cytosolic MEF2C is localized in the mitochondria of motor neurons in the cortex and spinal cord of ALS patients and ALS mouse models [55].

However, it is not known whether there is a casual relationship between the enhancer mutation of *MEF2C* gene and the onset of brain disorders. Currently, it is well accepted that disruption of enhancer function through genetic mutation or chromosomal rearrangement is closely linked to disease-driving mechanisms [56]. Since we verified that the enhancer mutation of *MEF2C* down-regulates its own gene expression, we utilized *Mef2c*-KD mouse model to investigate the loss of *Mef2c* function in vivo. Interestingly, *Mef2c*-KD mice exhibited dystonia-like phenotype and reduction of hindlimb and forelimb muscle strength. Moreover, the longitudinal behavior analyses showed a progressive dysfunction of motor coordination in *Mef2c*-KD mice that is consistent with the behavioral phenotypes of ALS mice [57–60]. Building upon previous findings and our own data, we propose that loss of MEF2C function by its enhancer mutation can be a risk factor for motor neuronal dysfunction in mice.

Previous studies have shown that ALS patients and ALS mice (SOD1-G93A) exhibit hyperexcitability of cortical motor neurons, but the mechanism of hyperexcitability is not known [61, 62]. Our electrophysiology data shows a significant increase in intrinsic excitability by increasing the number of action potentials in pyramidal neurons at cortical layer V of *Mef2c*-deficient mice without affecting the threshold for firing action potentials. Consistent with our data, Wainger et al. reported hyperexcitability of SOD1 ALS-derived motor neurons and no changes in the threshold of action potentials [61]. Once the higher frequency of firing leads to accumulation of Na^+^, Ca^2+^, and Cl^−^ in neuronal cytosol and release of K^+^ to the extracellular space [63], it can cause excitotoxicity, ultimately leading to neuronal damage [64, 65]. Additionally, given that mitochondria activity is pivotal for modulating the excitability of motor neuron, MEF2C deficit-associated mitochondrial dysfunction can lead to a pathological hyperexcitability and motor neuronal damage [66].

## Conclusions

In conclusion, we identified an ALS-associated SNP, rs304152, located in the distal enhancer of *MEF2C* and verified its mechanistic role as an epigenetic modulator for the transcription of its own gene. The loss of MEF2C function due to the rs304152-G allele is associated with metabolic disruptions in motor neurons. This finding suggests that, in part, MEF2C dysregulation caused by the rs304152-G allele may contribute to motor neuronal degeneration in the pathogenesis of ALS. Considering the possibility of altered splicing due to the intronic rs304152 mutation, analysis with the SpliceAl tool predicted no splicing changes for this variant [67, 68]. Moreover, no *MEF2C* splicing pattern has been associated with ALS to date. Thus, the potential impact of *MEF2C* splicing on neurodegeneration remains to be clarified in future studies. In this study, we focused on a mechanism suggesting that the rs304152 mutation reduces *MEF2C* gene expression by disrupting ATF4 binding to the *MEF2C* enhancer region. The longitudinal behavioral study further indicates that *Mef2c* deficiency causes progressive muscle weakness and impairment of motor coordination skills in mice similar to ALS mice phenotypes. Together, this study proves that the noncoding variant of *MEF2C*, rs304152, is a *bona fide* risk factor of motor neuron disease, and provides an insight on how a noncoding genetic mutation drives epigenetic changes in the pathogenesis of motor neuron disease.

## Electronic supplementary material

Below is the link to the electronic supplementary material.

Supplementary Material 1

## Data Availability

All supporting information and data are available in the article and supplementary files.

## Abbreviations

MEF2C: Myocyte Enhancer Factor 2 C
MEF2C-KD: MEF2C-Knockdown
TEM: Transmission Electron Microscopy
ALS: Amyotrophic Lateral Sclerosis
GWAS: Genome-Wide Association Study
SNP: Single-Nucleotide Polymorphism
CNN: Convolutional Neural Network
SCZ: Schizophrenia
TFBS: Transcription Factor Binding Site
LD: Linkage Disequilibrium
MEF2C-O/E: MEF2C Overexpression
TFAM: Mitochondrial Transcription Factor A
TOMM20: Translocase of the Outer Mitochondrial Membrane 20
MFN1: Mitofusin 1
DRP1: Dynamin-Related Protein 1
mtDNA: Mitochondrial DNA
