# Supplementary material for: Loss of MEF2C function by enhancer mutation leads to neuronal mitochondria dysfunction and motor deficits in mice
Source: Mol Neurodegener. 2025 Feb 7;20:16. doi: 10.1186/s13024-024-00792-y (PMC11806887; doi:10.1186/s13024-024-00792-y)
Supplement: Supplementary file 1 — Supplementary Material 1 [file 13024_2024_792_MOESM1_ESM.docx]

**Supplementary Material & Data**

Loss of *MEF2C* function by enhancer mutation leads to neuronal mitochondria dysfunction and motor deficits in mice

Ali Yousefian-Jazi^1^, Suhyun Kim^1^, Jiyeon Chu^1,2^, Seung-Hye Choi^1^, Phuong Thi-Thanh Nguyen^1,3^, Uiyeol Park^1,4^, Min-gyeong Kim^3,5^, Hongik Hwang^6^, Kyungeun Lee^7^, Yeyun Kim^1,2^,

Seung Jae Hyeon^1^, Hyewhon Rhim^5^, Hannah L. Ryu^8^, Grewo Lim^8,9^, Thor D. Stein^8,9^,

Kayeong Lim^5^, Hoon Ryu^1,3,10*^, and Junghee Lee^8,9*^

^1^Laboratory for Brain Gene Regulation and Epigenetics, Brain Science Institute, Korea Institute of Science and Technology (KIST), Seoul 02792, Republic of Korea

^2^Department of Integrated Biomedical and Life Science, College of Health Science, Korea University, Seoul 02841, Republic of Korea

^3^KIST School, Division of Bio-Medical Science & Technology, University of Science and Technology (UST), Seoul 02792, Republic of Korea

^4^Department of Biochemistry & Molecular Biology, College of Medicine, Hanyang University, Seoul 04763, Republic of Korea

^5^Brain Science Institute, Korea Institute of Science and Technology (KIST), Seoul 02792, Republic of Korea

^6^Department of Life Science, University of Seoul, Seoul 02504, Republic of Korea

^7^Advanced Analysis Data Center, Korea Institute of Science and Technology (KIST), Seoul 02792, Republic of Korea

^8^Boston University Alzheimer’s Disease Research Center and Department of Neurology, Boston University Chobanian & Avedisian School of Medicine, Boston, MA 02118, USA

^9^VA Boston Healthcare System, Boston, MA 02130, USA

^10^ KHU-KIST Department of Converging Science and Technology, Kyung Hee University, Seoul 02447, Republic of Korea

Correspondence to: Junghee Lee and Hoon Ryu

Laboratory for Brain Gene Regulation and Epigenetics, Brain Science Institute, Korea Institute of Science and Technology (KIST), Seoul 02792, South Korea

[hoonryu@kist.re.kr](mailto:hoonryu@kist.re.kr)

Department of Neurology, Boston University Chobanian & Avedisian School of Medicine, Boston, MA 02118, USA

[junghee@bu.edu](mailto:junghee@bu.edu)

**Materials and methods**

**Animals**

The C57BL/6 mice were used for virus injections, and transgenic ALS mice (G93A) were used for immunohistochemistry. Male transgenic ALS mice of the G93A H1 high-expresser strain (Jackson Laboratories, Bar Harbor, Maine) were bred with females with a similar background (B6/SJLF1). Offspring were genotyped using a PCR assay on tail DNA. All mice were kept under a 12:12-h light-dark cycle (lights on at 8:00 AM) and has ad libitum access to food and water. The animal study was conducted following the guide for the care and use of laboratory animals and was approved by KIST Animal Care Committees (Animal Protocol Approval Number: KIST-IACUC-2024-001). Immunohistochemistry and behavioral tests were performed on virus-injected mice, utilizing both sexes of 10- to 12-month-old wild-type littermates. In this study, the investigators involved in the care and assessment of the animals were blinded to the group allocations and outcome analyses.

**NSC-34 cell culture**

Motor neuron-like hybrid cell lines (NSC-34) were cultured and sub-cultured on collagen type-I-coated culture dishes and grown in Dulbecco’s modified Eagle’s medium (Invitrogen, San Diego, CA, USA) supplemented with 10 % fetal bovine serum (HyClone, USA), containing 100 U/mL penicillin and 100 µg/mL streptomycin (Invitrogen) [1, 2]. Cultures were used 10 to 15 passages. The cells were incubated under conditions of 5% CO^2^/95% air at 37 °C described in an earlier study [3]. Motor neuron-like hybrid cell lines (NSC-34) were transfected with the pCMV-SPORT6-ATF4 and pCMV3-C-GFPDpark-MEF2C vectors, and infected with the pAAV-hSyn(pro)-shMEF2C-GFP and AAV-MEF2C-mCherry viruses according to the method explained in a previous study [4].

**Real-time qPCR**

Total RNA was isolated from transiently transfected cells using an RNeasy Plus Mini Kit (QIAGEN, USA), cDNA was synthesized using a Maxime RT PreMix Kit (iNtRON Biotechnology, Korea), and subjected to quantitative PCR analysis using SYBER green master mix (Invitrogen, USA). For qPCR, we used ABI PRISM 7700 Sequence Detection System Instrument and software (Applied Biosystems, Foster City, CA, USA) with manufacturer's recommended conditions. The amplification factor was calculated by comparative threshold cycle (Ct) method, and the mitochondria-encoded genes (*Nd2*, *Nd4*, *Nd5*, *16srRNA*) were normalized to *12SrRNA*, while *Tfam* and *Tomm20* were normalized to *Gapdh* levels in parallel reactions. Information on the qPCR primers is provided in Supplementary Table 5.

**Chromatin Immunoprecipitation (ChIP) and quantitative PCR assay**

ChIP for H3K9me3 binding to DNA was performed using a ChIP assay kit (Chem Cruz, USA). NSC-34 cells infected by shControl and shMef2c viruses were crossed-linked with 1% formaldehyde for 10 mins at room temperature. Lysates were sonicated for 30s in 30 cycles using a Bioruptor (Diagenode Inc., NJ, USA). The supernatant was diluted in ChIP dilution buffer after centrifugation and incubated overnight at 4 °C with anti-ATF4 or anti-MEF2C antibody. Then, 50μl of magnetic bead were added and incubated at 4 °C for 2hrs with rotation. Beads were pelleted and washed with high salt and wash buffer 3 times. After that, we eluted Immune complexes by incubation with 500μl of fresh elution buffer (1% SDS, 0.1 M NaHCO3) and 20μl of 5 M NaCl. We added 500mM EDTA, 1 M Tris and proteinase K to the eluate and incubated for 1hr at 45°C. DNA was recovered by phenol, chloroform and isoamyl and precipitates with 100% ethanol. After precipitation, the pellet was washed with 70% ethanol and dissolved in 20μl DW. The qPCR primers information is described in Supplementary Table 6.

**Measurement of mitochondrial membrane potential and oxidative stress**

MitoTracker (Thermo Scientific, USA) and MitoSox (Thermo Scientific, USA) staining (CMS-ROS) were performed to measure the mitochondrial membrane potential and oxidative stress, respectively, in motor neuronal cell line [5]. The cells were stained with MitoTracker-Red (0.1 μM), MitoTracker-Green (0.1 μM), MitoSox-Red (2.5 μM) or MitoSox-Green (2.5 μM) for 30 mins prior to fixation and subjected to DAPI (Sigma, USA) staining. The intensity of mitochondrial membrane potential and oxidative stress was analyzed using the Fiji ImageJ program (NIH, USA).

**Cell viability assay**

Cell viability of motor neuron cells after infection with *MEF2C*-KD virus was assessed using the MTS [3-(4, 5-dimethyldiazol-2-yl)-5-(3-carboxy-methoxyphenyl)-2-(4-sulfophenyl)-2H-tetazolium inner salt] assay (PMS, Promega Corp., USA). NSC-34 cells were seeded into a 24-well plate at a density of 4×10^4^ cells per well. The virus was then infected to the cells after 16hrs of stabilization, and the viability of the cells was determined after 24, 48 and 72hrs using CellTiterGlo reagent (G7572, Promega, USA). A standard curve was fitted, and GI50 values were calculated using Graphpad prism 9.0 software (GraphPad Software, USA). All assays were performed in triplicate, and the mean standard error was determined from three independent experiments.

**Immunocytochemistry**

ATF4, MEF2C, Cytochrome c, TFAM and active caspase-3 (Cas-3) immunoreactivity were determined in the motor neuron cell line. For immunostaining in cultured cells, the cells were rinsed with PBS twice and fixed with 4% PFA for 10 mins at room temperature. Fixed cells were incubated for 30 mins in a blocking solution (2% donkey/goat serum, 0.3% Triton-X100 in 0.1 M PBS) and were further incubated with anti-ATF4 (1:200, ab216839, Abcam, USA), anti-MEF2C (1:100, MBS1498383, MyBioSource, USA), anti-mtTFA (1:100, ab272885, Abcam, USA), anti-cytochrome c (1:100, sc-13560, Santa Cruz, USA) and anti-cleaved caspase-3 (1:200; Cell Signaling, Danvers, MA, USA) at 4 ◦C for 24hrs. Photo-images were taken by using an Olympus epifluorescence microscopy (Olympus, Japan). The semi-quantitative analysis of immunoreactivity was performed by the Fiji ImageJ program.

**Western blot analysis**

Western blot analyses were performed as described previously [6]. The transferred blots were incubated with anti-MEF2C (MBS1498383, MyBioSource, USA), anti-LaminB1 (ab16048, Abcam, USA) and anti-COX4 (ab16056, Abcam, USA) as primary antibodies at 4 °C for 24hrs. After washing 3 times with Tris-buffered saline with 0.05% Tween 20, the blots were incubated with the appropriate secondary antibodies conjugated to horseradish peroxidase (HRP; anti-rabbit HRP (#1706515 and #1706516, NIF 824, Amersham Pharmacia; Pierce, USA) at room temperature for 2hrs. Then, the blots were developed by Immobilon Western ECL solution (#WBKLS0500; Merck Millipore, USA) and immunoreactivity bands were visualized using an Image Station 4000MM (#745280; Kodak, Japan).

**Immunohistochemistry**

 After anesthetized the mice with 2% avertin (20mgg−1, intraperitoneally), the mice perfused with 0.9% saline followed by ice-cold 4% paraformaldehyde (PFA, Sigma, USA). The brains and spinal cords were post-fixed overnight at 4°C in 4% PFA and then dehydrated in 15% and 30% sucrose for 48hrs. Coronal cortical sections were cut at 30μm using a cryostat and stored in a storage solution at 4°C. The sections were washed in PBS and incubated for 1hr in a blocking solution (0.3% Triton X-100, 2% normal donkey serum in 0.1M of PBS). Primary antibodies in the blocking solution were immunostained on a shaker at 4°C overnight. Then, the sections were incubated with secondary antibodies for 1hr at room temperature. The sections washed 3 times in PBS before and after the second antibodies incubation. DAPI staining was performed by adding DAPI solution (1:5,000; Abcam, USA) during the second washing step. Finally, the sections were mounted with fluorescent mounting medium (Dako, USA) and dried at room temperature. A series of fluorescent images were obtained with a Nikon A1 confocal microscope (Nikon, Japan) with 26 μm Z stack images in 2 μm steps processed using the NIS-Elements software (ver. 4.5, Nikon, Japan) and ImageJ software (ver. 1.52s, NIH, USA). The dilution amounts for primary antibodies were as follows: anti-MEF2C (1:100, MBS1498383, MyBioSource), anti-ND4 (1:200, PA5-116791, Thermo Fisher Scientific), anti-CTIP2 (1:100, ab240636), anti-DRP1 (1:100, sc-21804), anti-TDP-43 (1:200, A19123, ABclonal), and anti-TOMM20 (1:100, sc-17764). Secondary antibodies were diluted 1:500 in the blocking solution for 2hrs at room temperature.

**Subcellular fractionation and isolation of mitochondria**

Mitochondria were isolated from the forebrain part minus the olfactory bulbs of C57BL/6 mouse brain tissue by sucrose density gradient centrifugation as explained previously [7, 8].

**AAV-virus production and stereotaxis injection**

To produce high titer AAV (1 x 10^9^~10^11^ pfu/ml), the target plasmid, pRC, and pHelper plasmids (Addgene, USA), were transiently transfected into HEK293TN cells. Cell lysates were harvested at 72 hours after transfection and treated with benzonase (50 unit/ml; Sigma, USA). Virus particles were purified and concentrated with a heparin column (GE healthcare, Sweden) in 100k filtering tube (Millipore, USA). pAAV-hSyn(pro)-shMef2c-GFP virus was injected to five C57BL/6 mice (10 months old) using a stereotaxic micro-injector (Stoelting Co., USA) with a microsyringe pump (Micro 4, WPI, USA) and a calibrated 50μl Hamilton syringe (Hamilton co., USA) fitted with a 33-gauge needle (WPI, USA) (0.1 μl/min) in the motor cortex layer V (AP; ± 0.5 mm, ML; -1 mm, DV; -1.4 mm). Control groups of 5 mice with the same age of cases were injected with the pAAV-hSyn(pro)-shControl-GFP virus. The sequence information of shRNA-*Mef2c* is described in Supplementary Table 7. Intrathecal injection was performed by lumbar puncture. One group was injected with pAAV-hSyn(pro)-shControl-GFP and the other group was injected with pAAV-hSyn(pro)-shMef2c-GFP. The mice were injected by inserting the needle between the groove of L4 and L5 vertebrae and observing for a tail flick as the sign of successful entry of the needle in the intradural space. Then, we injected the 6 μl of virus into each mouse. Once injection was performed, the mouse was moved back to the cage to recover from anesthesia. All mice were injected at the age of 10 months.

**Behavioral tests**

The behavioral tests were performed in a longitudinal behavioral study at 3, 6, 9 and 12 weeks after injection. **Open field test:** To measure general motor function and exploratory activity, we used an open field test. The mice activity monitors in an open field arena (40 × 40 cm^2^; height of walls, 40 cm) equipped with a camera at the top. The floor of the arena was made of white plastic. Animals were released in the corner of the arena and monitored for 10 mins. Distance traveled, velocity, and time spent in the corners (6 × 6 cm) and in the central zone (18 × 18 cm) were measured using EthoVision XT software (version 13, Noldus, USA). **Cylinder test:** Spontaneous movement was assessed by placing mice in a small transparent cylinder (height 15.5 cm; diameter 12.7 cm) [9]. Spontaneous activity was videotaped using 2 cameras for 3 mins. The number of rears, grooming and total grooming duration were measured for WT and AAV-shMEF2C injected mice. Videotapes were viewed and rated in slow motion by an experimenter blind to the mouse genotype. A rear was counted when an animal made a vertical movement with both forelimbs removed from the ground. **Tail suspension test:** Limb movement was assessed by the tail suspension test. Briefly, the mouse’s ventral posture was videotaped while the mouse was suspended by the tail for 10 seconds, followed by a touchdown and subsequent suspension for 20 more seconds (in total 30 seconds of tail suspension). The number of forelimb and hindlimb clasping were counted through slow-speed video monitoring. Coordinate plots were drawn to check the altered hindlimb clasping behavior by labeling the tail base and hindlimbs using deep learning framework, DeepLabCut, in the first 10 seconds of suspension [10]. **Accelerating** **wheel test:** In this test, the mouse is placed inside of the wheel and the rotation speed is accelerated from 4 to 15 rpm in 3 min. The training phase is the first 3 min of wheel running with the same conditions the real test right before the test. The testing phase is recorded by 2 cameras from bottom and front. For the footprint analysis, the hindlimbs and forelimbs of mice were dyed with different colors using a non-toxic animal marking stick (MS Schippers, AH Bladel) 4hrs before the test. Gait analysis was simulated by detecting the colored hindlimb using the EthoVision XT software (Noldus, version 13, USA). Five or six strides with continuous ambulatory movement were analyzed for each mouse[11]. **Inverted grid test:** This was a test of muscular strength for all four limbs [12]. The grid screen is a 23×39 cm rectangle consisting of a wooden frame and 12 mm squares of 1 mm diameter wire. The mouse is placed in the center of the screen, then it is inverted slowly. The test is recorded by a camera from the top, and the time it took for the mouse to fall is measured.

**Acute slice preparation**

The 10-month-old C57BL/6 mice injected with either shControl or shMEF2C viruses in the motor cortex layer V were briefly anesthetized with halothane. After confirming the adequate depth of anesthetic by the lack of a pedal withdrawal reflex, a mouse was quickly decapitated, and the brain was isolated. Acute brain slices (coronal section, 300-μm thick) were prepared using a vibratome (Leica, VT1000S) in an ice-cold cutting buffer containing (in mM) 234 sucrose, 2.5 KCl, 1.25 NaH_2_PO_4_, 24 NaHCO_3_, 11 glucose, 0.5 CaCl_2_, 10 MgSO_4_, saturated with 95% O_2_ and 5% CO_2_. Subsequently, the slices were recovered in a recovery artificial cerebrospinal fluid (aCSF) solution containing (in mM) 124 NaCl, 3 KCl, 1.25 NaH_2_PO_4_, 26 NaHCO_3_, 10 glucose, 6.5 MgSO_4_, 1 CaCl_2_, saturated with 95% O_2_ and 5% CO_2_ at 35 °C for an hour, and then maintained at room temperature throughout the experiments.

**Electrophysiology**

Following the recovery, the brain slice was transferred to a recording chamber and continuously perfused with a recording aCSF solution containing (in mM) 124 NaCl, 3 KCl, 1.25 NaH_2_PO_4_, 26 NaHCO_3_, 10 glucose, 1.3 MgSO_4_, 2.5 CaCl_2_, saturated with 95% O_2_ and 5% CO_2_ at room temperature. The recording aCSF was supplemented with 10 μM DNQX, 50 μM D-APV and 100 μM picrotoxin to inhibit synaptic transmission. Motor cortex layer V pyramidal neurons infected with shControl or shMEF2C were visualized under a microscope (Olympus, BX51WI, Japan) equipped with a CCD camera (Hamamatsu, C3077, Japan). To examine intrinsic neuronal excitability, GFP-positive motor cortex layer V pyramidal neurons were whole-cell patched using a glass pipette with 5-7 MΩ resistance under voltage clamp configuration, and the neurons were initially held at - 65 mV. The recording mode was then switched to current clamp configuration, and a series of current injection ranging from 0 to 330 pA (30-pA step, 12 steps) were given. The internal solution contained (in mM) 130 K-gluconate, 10 KCl, 10 HEPES, 0.2 EGTA, 4 ATP-Mg, 0.5 GTP-Na_2_, 10 phosphocreatine-Na_2_ (pH = 7.25 and osmolality = 290 mOsm). All data were collected using a MultiClamp 700B amplifier (Molecular Devices) digitized at 10kHz with a Digidata 1550 digitizer (Molecular Devices). pClamp 10 software (Molecular Devices, USA) was used for data acquisition and analysis.

**Transmission electron microscopy (TEM)**

The mouse brain samples were fixed for 1hr in a mixture of 2% glutaraldehyde, 0.2% freshly prepared tannic acid, and 0.1 M sodium cacodylate (pH 7.4). After washing in cacodylate, they were post-fixed in 0.5% OsO_4_, and embedded in Durcupan (Fluka, Switzerland) [13]. Thin sections were prepared with uranyl acetate and lead citrate contrast and examined by a Jeol CX 100 electron microscope. For immune-gold labelling, mouse cortex sections were incubated with anti-MEF2C (MBS1498383, MyBioSource, USA) polyclonal antibody in blocking solution (0.5% Ttiton-X100 with 5% NGS in PBS) and then proceeded with goat anti-rabbit antibody conjugated with gold particles (G3779, 10 nm, Sigma, USA).

**Statistical analysis**

Data are presented as the mean ± SEM. For behavioral analysis, Ethovision XT (Noldus, USA) was used. For image analysis, ImageJ software (NIH, USA) and IMARIS software (Oxford instrument, UK) were used. All statistical analyses were performed using Prism v.8.4.3 (GraphPad Software, USA). An unpaired, two-tailed Student’s t-test and repeated measures ANOVA were used to compare two groups and more than two groups, respectively. For immunocytochemistry quantification analysis, the linear mixed model (LMM) was used to evaluate statistical significance between two groups with nested or repeated data [14]. Statistical differences were considered significant when P < 0.05 and the significance was set at **P* < 0.05, ***P* < 0.01, ****P* < 0.001, that are indicated in the figures or figure legends. No statistical methods were used to predetermine sample sizes, and sample sizes were similar to those reported in previous publications.

**References**

1. Ryu H, Jeon GS, Cashman NR, Kowall NW, Lee J: **Differential expression of c-Ret in motor neurons versus non-neuronal cells is linked to the pathogenesis of ALS**. *Laboratory Investigation* 2011, **91**(3):342-352.

2. Cashman NR, Durham HD, Blusztajn JK, Oda K, Tabira T, Shaw IT, Dahrouge S, Antel JP: **Neuroblastoma × spinal cord (NSC) hybrid cell lines resemble developing motor neurons**. *Developmental Dynamics* 1992, **194**(3):209-221.

3. Löscher W: **Pharmacological effects and mechanisms of action**. In: *Valproate.* Edited by Löscher W. Basel: Birkhäuser Basel; 1999: 7-45.

4. Lee J, Ryu H, Kowall NW: **Motor neuronal protection by L-arginine prolongs survival of mutant SOD1 (G93A) ALS mice**. *Biochem Biophys Res Commun* 2009, **384**(4):524-529.

5. Hyeon SJ, Park J, Yoo J, Kim SH, Hwang YJ, Kim SC, Liu T, Shim HS, Kim Y, Cho Y *et al*: **Dysfunction of X-linked inhibitor of apoptosis protein (XIAP) triggers neuropathological processes via altered p53 activity in Huntington's disease**. *Prog Neurobiol* 2021, **204**:102110.

6. Choi SH, Yousefian-Jazi A, Hyeon SJ, Nguyen PTT, Chu J, Kim S, Kim S, Ryu HL, Kowall NW, Ryu H *et al*: **Modulation of histone H3K4 dimethylation by spermidine ameliorates motor neuron survival and neuropathology in a mouse model of ALS**. *J Biomed Sci* 2022, **29**(1):106.

7. Lai JC, Clark JB: **Preparation of synaptic and nonsynaptic mitochondria from mammalian brain**. *Methods Enzymol* 1979, **55**:51-60.

8. Ryu H, Lee J, Impey S, Ratan RR, Ferrante RJ: **Antioxidants modulate mitochondrial PKA and increase CREB binding to D-loop DNA of the mitochondrial genome in neurons**. *Proceedings of the National Academy of Sciences* 2005, **102**(39):13915-13920.

9. Lee J, Hwang YJ, Kim Y, Lee MY, Hyeon SJ, Lee S, Kim DH, Jang SJ, Im H, Min SJ *et al*: **Remodeling of heterochromatin structure slows neuropathological progression and prolongs survival in an animal model of Huntington's disease**. *Acta Neuropathol* 2017, **134**(5):729-748.

10. Mathis A, Mamidanna P, Cury KM, Abe T, Murthy VN, Mathis MW, Bethge M: **DeepLabCut: markerless pose estimation of user-defined body parts with deep learning**. *Nature Neuroscience* 2018, **21**(9):1281-1289.

11. Heikkinen T, Bragge T, Bhattarai N, Parkkari T, Puoliväli J, Kontkanen O, Sweeney P, Park LC, Munoz-Sanjuan I: **Rapid and robust patterns of spontaneous locomotor deficits in mouse models of Huntington's disease**. *PLoS One* 2020, **15**(12):e0243052.

12. Kondziella W: **A New Method For The Measurement Of Muscle Relaxation In White Mice**. *Arch Int Pharmacodyn Ther* 1964, **152**:277-284.

13. Lee J, Kim Y, Liu T, Hwang YJ, Hyeon SJ, Im H, Lee K, Alvarez VE, McKee AC, Um S-J *et al*: **SIRT3 deregulation is linked to mitochondrial dysfunction in Alzheimer's disease**. *Aging Cell* 2018, **17**(1):e12679.

14. Ahmadpour N, Kantroo M, Stobart MJ, Meza-Resillas J, Shabanipour S, Parra-Nuñez J, Salamovska T, Muzaleva A, O’Hara F, Erickson D *et al*: **Cortical astrocyte N-methyl-D-aspartate receptors influence whisker barrel activity and sensory discrimination in mice**. *Nature Communications* 2024, **15**(1):1571.

15. Yang D, Jang I, Choi J, Kim M-S, Lee AJ, Kim H, Eom J, Kim D, Jung I, Lee B: **3DIV: A 3D-genome Interaction Viewer and database**. *Nucleic Acids Research* 2017, **46**(D1):D52-D57.


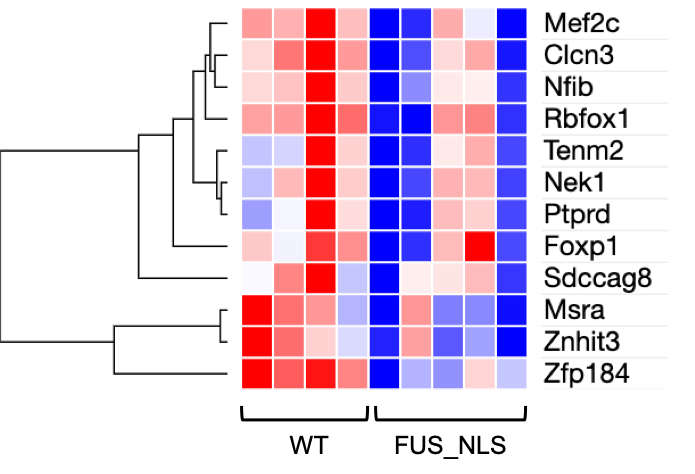


**Supplementary Fig. 1.** Heatmap analysis for selected 12 genes by CNN algorithm in frontal cortex mRNA transcriptome data from 22-month-old FUS-NLS male mice (25).

**Supplementary Fig. 2.** **MEF2C immunoreactivity is decreased in cortical layer V pyramidal neurons and spinal cord motor neurons of ALS patients and ALS (G93A) mice. (A)** DAB staining images show the MEF2C immunoreactivity in pyramidal neurons of the cortical layer V in the motor cortex in normal subjects (*N* = 5) and ALS patients (*N* = 5). **(B)** DAB staining images show the MEF2C immunoreactivity in motor neurons of the spinal cord ventral horn in normal subject (*N* = 3) and ALS patients (*N* = 3). Scale bars: 5 μm. Right panels show densitometry results of MEF2C immunoreactivity in both cortical layer V pyramidal neurons and spinal cord motor neurons, respectively. A total of 30 cells/group were counted (6 cells/case) in cortex and 24 cells/group were counted (8 cells/case) in spinal cord. Statistics were calculated using Student's t-test, *****, *P* < 0.001, ***, *P* = 0.05. **(C)** DAB staining images show MEF2C immunoreactivity in cortical layer V pyramidal neurons of ALS (G93A) mice (*N* = 5) and WT mice (*N* = 5). **(D)** DAB staining images show MEF2C immunoreactivity in spinal cord motor neurons of ALS (G93A) mice (*N* = 5) and WT mice (*N* = 5). Right panels show the quantitation of MEF2C immunoreactivity level in both cortical layer V pyramidal neurons and spinal cord motor neurons, respectively. A total of 30 cells/group were counted (6 cells/mouse). Statistics were calculated using Student's t-test, *****, *P* < 0.001, ***, *P* = 0.026. The nuclei were counterstained with hematoxylin. Scale bars: 5 μm. Error bars represent means ± SEM.


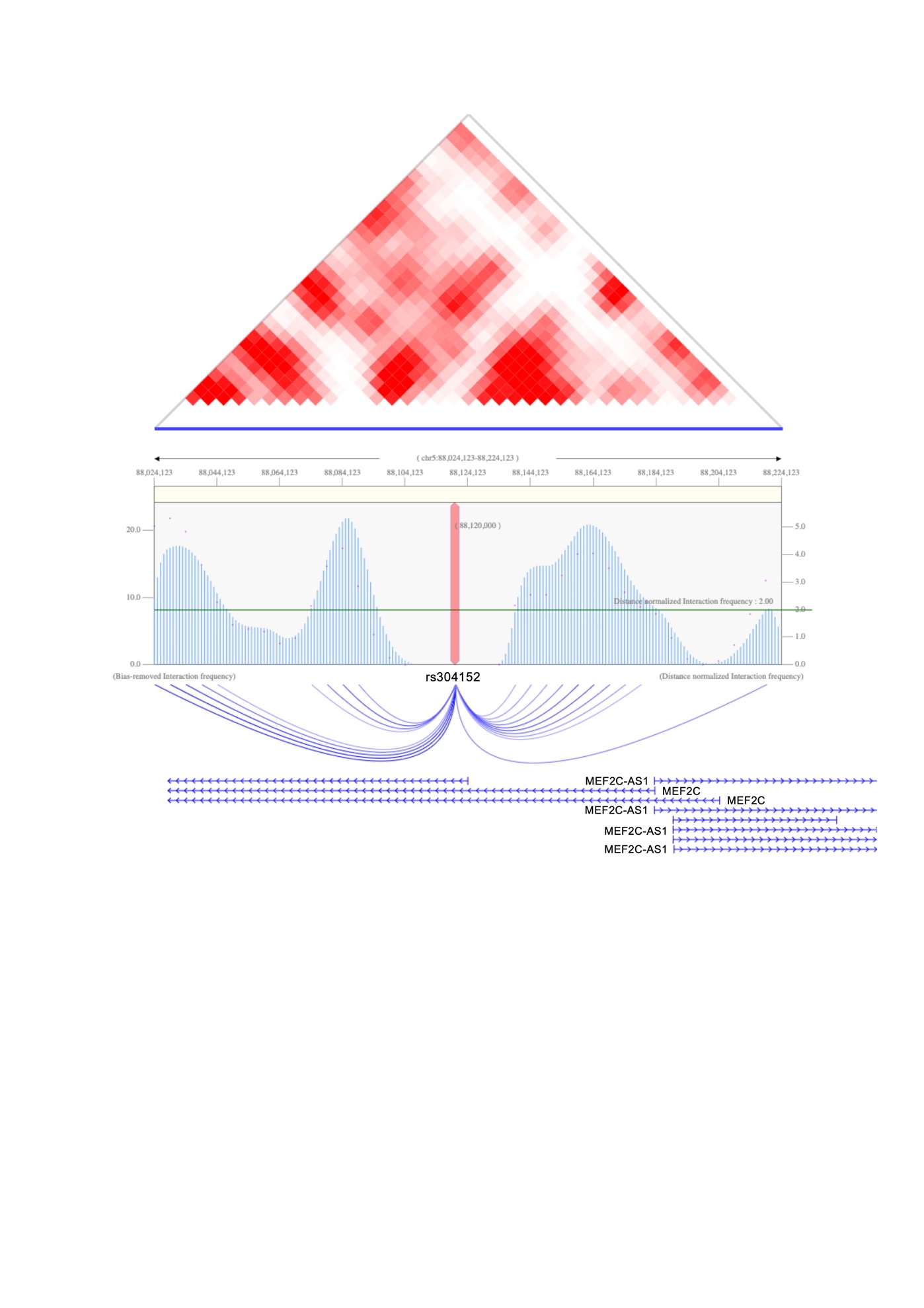


**Supplementary Fig. 3.** **Hi‐C data showed the long‐range interactions between rs304152 and *MEF2C* promoter in human prefrontal cortex.** The TAD result and interaction peaks of rs304152 and nearby genes within a 100 kb region using Hi-C data across human prefrontal cortex. Data from the 3DIV database[15].

**Supplementary Fig. 4.** **(A)** Schematic representation of the constructs used in the luciferase reporter assays. Right panel shows the construct containing the rs304152-G allele (MT) showed less luciferase activity than the construct containing the rs304152-T allele (WT). The experiment was repeated three times. Statistics were calculated using Two-way ANOVA (*n* = 4/group, *****, *P* < 0.001). Error bars represent means ± SEM. **(B)** Schematic representation of the constructs used in the luciferase reporter assays. Right panel shows the reporter activity for the construct containing the rs304152-T allele (WT) increased approximately 1.4-fold in response to tunicamycin, whereas no significant change was observed for the construct containing the rs304152-G allele (MT). **(C)** DNA sequencing and chromatograms show that ATF4 ChIP DNA samples from *MEF2C* enhancer region contains duplex of WT or MT form. Alignment was generated by SnapGene software (GSL Biotech; available at snapgene.com).

**Supplementary Fig. 5. The rs304152 reduces the expression of nuclear-encoded MEF2C target genes in the frontal cortex. (A)** A schematic representation of the process for identifying downregulated genes in *Mef2c*-cKO mice that are also downregulated in *Fus* Knock-in mice. **(B)** Heatmap analysis of four selected genes and three nuclear-encoded mitochondria-associated genes in frontal cortex mRNA transcriptome data from 22-month-old FUS-NLS male mice (25). **(C)** Violin plots of *MYO16*, *NRIP3,* and *NTNG1,* and **(D)** *MFN1*, *TOMM20,* and *TFAM* normalized expression levels according to alleles of rs304152 in the frontal cortex. The data were extracted from GTEX database.

**Supplementary Fig. 6.** **MEF2C localized to the mitochondria of pyramidal neurons in human postmortem brains and mouse motor cortex (layer V). (A)** Immunofluorescence staining of MEF2C (green) and TOMM20 (red), a mitochondria outer membrane marker, in cortical pyramidal neurons of human postmortem brains. Scale bars (white): 5 μm. Right panels show the number and ratio of MEF2C-positive signals in the nucleus and the mitochondria. A total of 28 cells/group were counted (7 cells/case) from *N* = 4 (3 cases are shown above and 1 case is derived from Main Figure 3C). **(B)** DAB staining images present MEF2C immunoreactivity in the cytosol of pyramidal neurons in the motor cortex (layer V) of normal subjects and ALS patients. The nuclei were counterstained with hematoxylin (blue). Scale bars: 5 μm. Right panel shows the percentile of MEF2C intensity in the nucleus versus the cytosol. A total of 50 cells/group were counted (10 cells/case) from *N* = 5 normal subjects, and *N* = 5 ALS patients. **(C)** Immunofluorescence staining of MEF2C (red) and TOMM20 (green) in the pyramidal neurons of mouse motor cortex (layer V). White dashed line indicates the colocalization analysis foci of MEF2C and TOMM20. Scale bars (white): 5 μm. Right panel exhibits the histogram of MEF2C and TOMM20 colocalization. **(D)** Full blots (developed with MEF2C, COX4 and LaminB1 antibodies) from Western blot analysis for the subcellular fractionation of mouse brain. **(E)** Western blot analysis for detecting MEF2C monomer and dimer from the subcellular fractions crosslinked with 1% glutaraldehyde.

**Supplementary Fig. 7. CRISPR-Cas9 cells derived *MEF2C* enhancer mutation exhibits less localization of MEF2C in mitochondria of HEK293T cells. (A)** Immunofluorescence staining of MEF2C (green) and MitoTracker (red) in CRISPR-Cas9-WT and -MT cells along with 3D reconstruction image made by Imaris 9 (Bitplane). Right: skeletonized images of mitochondria morphological structure by MitoTracker signals. **(B)** Mitochondria network size analysis performed by MiNA plugin in Fiji ImageJ software. A total of 10 cells/group were counted (5 cells/well) from *n* = 2 wells/group (WT, MT). Statistics were calculated using LMM (****, *P* = 0.001). **(C)** Quantitation of the number of MEF2C and MitoTracker colocalized voxels made by Imaris 9. A total of 15 cells/group were counted (5 cells/well) from *n* = 2 wells/group (WT, MT). Statistics were calculated using LMM (***, *P* = 0.018).

**Supplementary Fig. 8. The rs304152-G allele blocks the effect of UPR activation on ND4 induction. (A)** A schematic illustration of ATF4 and tunicamycin transfection for 24 hrs and 12 hrs, respectively, into HEK293T^CRISPR-Cas9^ cells containing the rs304152-T (WT) and rs304152-G (MT) alleles. **(B)** Immunofluorescence staining of ND4 in WT and MT cells transfected with ATF4 or tunicamycin. Scale bars (white): 5 μm. Right: Densitometry analysis shows that ND4 was induced in WT cells by ATF4 and tunicamycin, but there is no change in MT cells. A total of 28 cells/group were counted. Statistics were calculated using repeated measures ANOVA (***, *P* = 0.01, *****, *P* < 0.001).

**Supplementary Fig. 9.** **(A)** A schematic illustrating SH-SY5Y cells transfection with CMV-GFP, Cas9-NG, sgRNA, and ssODN plasmids. **(B)** Immunofluorescence staining of ND4 in GFP^+^ WT and MT cells. Right panel shows quantification of ND4 levels. A total of 15 cells/group were counted. Statistics were calculated using LMM (*****, *P* < 0.001). **(C)** Immunostaining of MitoSox (red) in GFP^+^ WT and MT cells. The nuclei were counterstained with DAPI (blue). Scale bars (white): 5 μm. Right panel shows quantification of MitoSox levels. A total of 15 cells/group were counted. Statistics were calculated using LMM (****, *P* = 0.003).


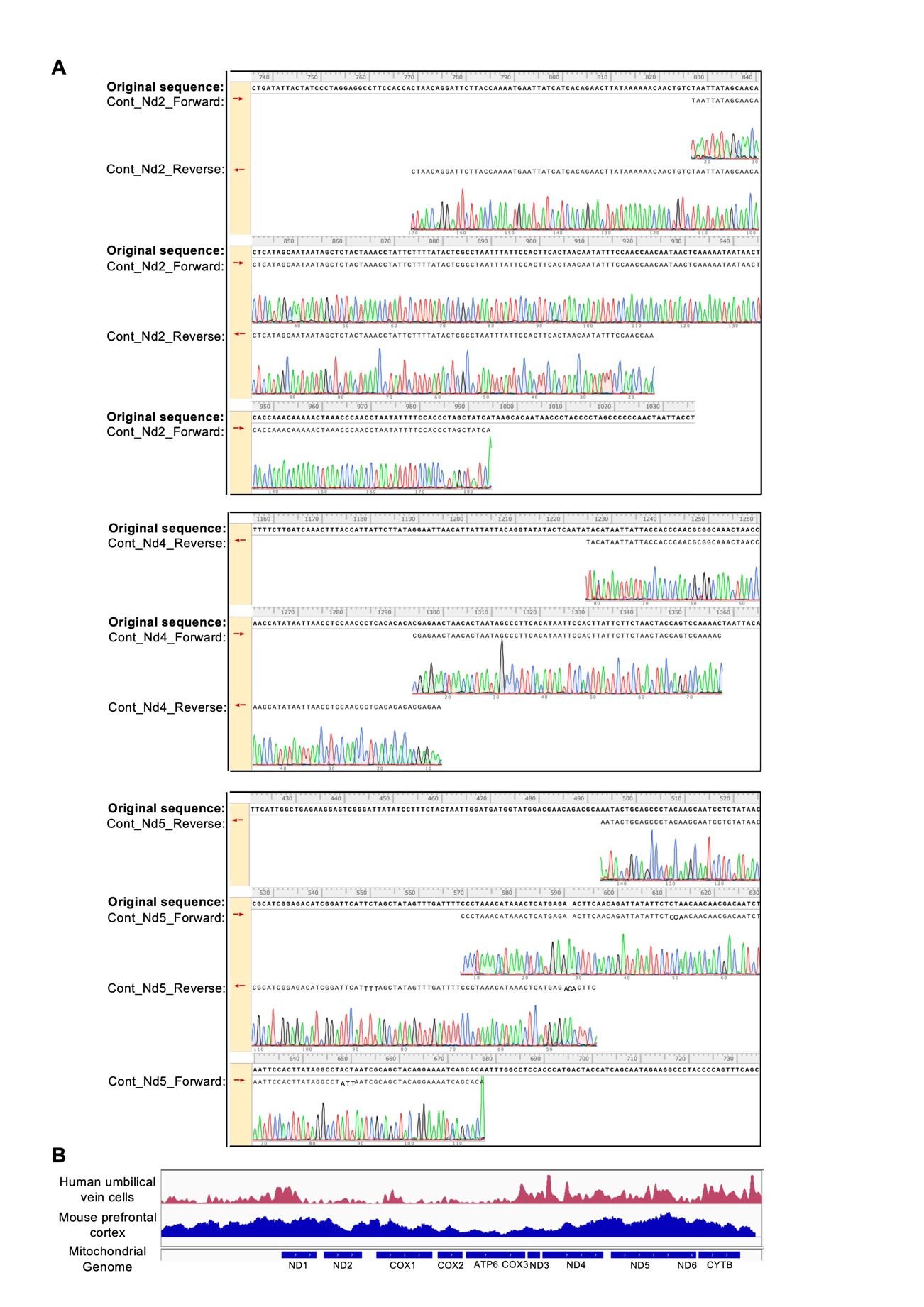


**Supplementary Fig. 10.** **(A)** DNA sequencing and chromatograms show that MEF2C-ChIP DNA samples from NSC-34 cell line contain mitochondrial *Nd2*, *ND4* and *ND5*. DNA sequences from MEF2C-ChIP were aligned with mouse mitochondrial genes, and alignment was generated by SnapGene software (GSL Biotech; available at snapgene.com). **(B)** Integrative Genome Viewer (IGV) peak reads of MEF2C in mitochondrial genomes in human umbilical vein cells (GSM809016) and mouse prefrontal cortex (GSM5244364). Alignment was generated by SnapGene software (GSL Biotech; available at snapgene.com).

**Supplementary Fig. 11. MEF2C binds to mitochondria DNA in the cortex of mouse brains.** **(A)** A scheme illustrating the procedure of nuclei and mitochondria fractionation by Ficoll density centrifugation from the cortex of four WT mice. MEF2C ChIP with subcellular fractions and quantification of MEF2C binding to mitochondria- versus nucleus-specific DNA by qPCR were performed sequentially. **(B)** Amplification (ΔCt) (top) and melting (bottom) curves show that DNA eluted from MEF2C ChIP for the mitochondria fraction is amplified with *Nd4*-specific primer but not with *Rgs6*-specific primer. **(C)** MEF2C-DNA occupancy with *Nd4*-specific primer is robustly detected in the mitochondria fraction but not in the nucleus fraction. **(D)** Amplification (ΔCt) (top) and melting (bottom) curves show that DNA eluted from MEF2C ChIP for the nucleus fraction is amplified with *Rgs6*-specific primer but not with *Nd4*-specific primer. **(E)** MEF2C-DNA occupancy with *Rgs6*-specific primer is robustly detected in the nucleus fraction but not in the mitochondria fraction. Data generated from 3 samples that were duplicated. Statistics were calculated using Student's t-test (*n* = 3/group, ****, *P* < 0.01). Error bars represent mean ± SEM. **(F)** Agarose gel electrophoresis confirmed that MEF2C-DNA occupancy with *Nd4*-specific primer is found in the mitochondria fraction while MEF2C-DNA occupancy with *Rgs6*-specific primer is found in the nucleus fraction. **(G)** Western blot analysis was performed to verify the purity of subcellular fractionations by specific antibodies for COX4, a mitochondria marker, and LaminB1, a nucleus marker (*n* = 4).

**Supplementary Fig. 12.** **MEF2C regulates nuclear- and mitochondrial-encoded genes in motor neuron (NSC-34) cells.** **(A)** A scheme illustrating MEF2C transfection in NSC-34 cells, RNA isolation and cDNA synthesis from 3 samples. **(B)** qPCR results show mRNA level alteration of mitochondria genes, *Nd2*, *Nd4*, *Nd5* and *12SrRNA* by MEF2C-O/E. Statistics were calculated using Student's t-test (*n* = 3/group, *Nd2*: ***, *P* = 0.05, *Nd4*: ***, *P* = 0.031). **(C)** A schematic illustrating dose-dependent shMef2c transfection (200 ng, 400 ng, and 800 ng per well) in NSC-34 cells. The graph in the right panel shows that *Nd4* mRNA levels are reduced by shMef2c in a dose-dependent manner. Statistics were calculated using One-way ANOVA (*n* = 4/group, *Nd2*: ***, *P* = 0.02, *Nd4*: ****, *P* = 0.003). **(D)** qPCR results show the mRNA levels of nuclear-encoded genes, including *Tfam*, *Tomm20*, *Mfn1*, and *DRP1*, in *Mef2c*-KD cells. Statistics were calculated using Student's t-test (*n* = 3/group, *Tfam*: ***, *P* = 0.048, *Tomm20*: ****, *P* = 0.007, *Mfn1*: ****, *P* = 0.003, *Drp1*: ***, *P* = 0.022). Immunocytochemistry showing **(E)** anti-TFAM and **(F)** anti-TOMM20 levels in mouse cortical primary neurons infected with the *Mef2c*-KD virus for 72 hrs. The right panels show the quantitative results of TFAM and TOMM20 levels in the cells cytosol. A total of 30 cells/group were counted (10 cells/well) from *n* = 3 wells/group (shControl, shMef2c). Statistics were calculated using LMM test (*****, *P* < 0.001). Scale bar (white): 5 μm **(G)** MEF2C-O/E in NSC-34 cells increased both MitoSox (green), and **(H)** MitoTracker signals (green). The nuclei were counterstained with DAPI (blue). Scale bars (white): 5 μm. Right panels are quantification of MitoSox and MitoTracker level in GFP^+^ cells. A total of 21 cells/group were counted (7 cells/well) from *n* = 3 wells/group (Control, MEF2C(O/E)). Statistics were calculated using LMM test (*****, *P* < 0.001). Error bars represent means ± SEM.

**Supplementary Fig. 13. The *MEF2C* enhancer region is not conserved in other species.** Alignment of the *MEF2C* enhancer sequence in humans, mice, canines, and primates.

**Supplementary Fig. 14. Verification of the efficiency of *Mef2c*-KD and *MEF2C*-O/E viruses in the NSC-34 cell line. (A)** NSC-34 cells were infected with shMef2c#1 (used in this study), shMef2c#2 or shControl for 48 hrs, and protein was extracted using RIPA buffer. Right panel shows Western blot quantification of MEF2C levels. *n* = 3 (shControl, shMef2c). Statistics were calculated using One-way ANOVA (****, *P* = 0.001). **(B)** qPCR results showing *Mef2c* mRNA levels in NSC-34 cells infected with AAV-MEF2C-mCherry (overexpression) and AAV-shMef2c-GFP (knockdown) viruses. *n* = 3 (shControl, shMef2c). Statistics were calculated using One-way ANOVA (****, *P* = 0.01). Error bars represent means ± SEM. Full blots developed with **(C)** MEF2C and **(D)** β-actin antibodies from the Western blot analysis shown in panel A.

**Supplementary Fig. 15.** **Decrease in** **Mef2c induces HS3ST2 expression in pyramidal neurons in cortical layer V.** **(A)** Immunostaining showing MEF2C levels at 9 weeks after delivery of the *Mef2c*-KD virus into cortical layer V. Scale bars (white): 5 μm. Right panel shows the quantitative results of MEF2C levels in GFP^+^ cells. A total of 30 cells/group were counted (6 cells/mouse) from *n* = 5 mice/group (shControl, shMef2c). Statistics were calculated using Student's t-test (*****, *P* < 0.001). **(B)** A schematic illustrating the datasets used to identify cortical pyramidal neuron layer V markers that are differentially expressed in *Mef2c*-cKO^Emx1^ mice. **(C)** Immunostaining of HS3ST2 and DAPI in shControl and shMef2c mice. Scale bars (white): 5 μm. Right panel shows densitometry analysis of HS3ST2 immunoreactivity levels in GFP^+^ cells. A total of 25 cells/group were counted (5 cells/mouse) from *N* = 5 mice/group (shControl, shMef2c). Statistics were calculated using Student's t-test (***, *P* = 0.012). Error bars represent means ± SEM.

**Supplementary Fig. 16. *Mef2c*-KD in the lumbar spinal cord leads to mitochondrial dysfunction and motor neuronal damage in mice. (A)** A scheme of intrathecal delivery of AAVs in mice (left panel). Detection of GFP and DAPI signals verified that AAV was delivered to the spinal cord ventral horn of mice (right panel). The immunostaining with **(B)** anti-MEF2C, **(C)** anti-ND4 and **(D)** anti-DRP1 after intrathecal injection of *Mef2c*-KD virus to mice. Scale bars (white): 5 μm. **(E)** Densitometry analysis showed the decrease of MEF2C and ND4 and increase of DRP1 immunoreactivity levels in GFP^+^ cells in *Mef2c*-KD mice. A total of 28 cells/group were counted (7 cells/mouse) from *N* = 4 mice/group (shControl, shMef2c). Statistics were calculated using Student's t-test (****, *P* = 0.002; *****, *P* < 0.001; ***, *P* = 0.044). **(F)** Immunofluorescence staining of TDP-43 and DAPI in shControl and shMef2c mice. Scale bars (white): 20 μm. Right: Densitometry analysis showed mislocalization of TDP-43 to the cytoplasm in lumbar spinal cord motor neurons of *Mef2c*-KD mice. A total of 16 cells/group were counted (4 cells/mouse) from *N* = 4 mice/group (shControl, shMef2c). **(G)** Lumbar spinal cord tissue sections were stained with cresyl violet. Scale bars: 20μm. Right: Densitometry analysis showed mislocalization of TDP-43 to the cytoplasm in motor neurons of the lumbar spinal cord in *Mef2c*-KD mice. A total of 44 cells/group were counted (11 cells/mouse) from *N* = 4 mice/group (shControl, shMef2c). Statistics were calculated using Student's t-test (****, *P* = 0.006). Error bars represent means ± SEM.

**Supplementary Fig. 17.** **Tail suspension and gait analysis of cortical layer V *Mef2c*-KD injected mice.** **(A)** Still images of tail suspension of shMef2c mice which showed abnormal hindlimb extension reflex compared to control mice. **(B)** The computer-assisted footprint in accelerated wheel running test.

**Supplementary Fig. 18.** **Open field test analysis results of cortical layer V *Mef2c*-KD injected mice.** **(A)** Comparing distance moved, cumulative duration and velocity of shControl and shMef2c mice in open-field test 3-, 6- and 9-weeks post-injection. Statistics were calculated using repeated measures ANOVA, *N* = 5 mice/group (shControl, shMef2c). Error bars represent means ± SEM. **(B)** Variable being measured in each case. Left shows track visualization used to obtain the total distance traveled of mouse (red shows the higher velocity). Right shows heatmap visualization.

**Supplementary Fig. 19.** **Longitudinal behavioral study for *Mef2c*-KD in the lumbar spinal cord of mice.** **(A)** A scheme illustrating intrathecal injection of *Mef2c*-KD virus at L4-L5 intervertebral space and performing behavioral study 3-, 6- and 9-weeks after injection. **(B)** Still images of representative sh*Mef2c* mouse exhibited hindlimb clasping posture in tail-suspension test. **(C)** Aggregated coordinate plots in the first 10 seconds of tail suspension of shControl and shMef2c representative mice at 3-weeks post-injection. **(D)** Forelimbs clasping frequency on tail suspension test at 3- and 6-weeks post-injection. Statistics were calculated using repeated measures ANOVA (*N* = 7 mice/group for shControl and *N* = 6 mice/group for shMef2c) (3weeks, ***, *P* = 0.017; 6weeks, ***, *P* = 0.047). **(E)** Minimal holding impulse in inverted grid test decreased for sh*Mef2c* mice. Statistics were calculated using repeated measures ANOVA (*N* = 7 mice/group for shControl and *N* = 6 mice/group for shMef2c) (3weeks, *P* = 0.409; 6weeks, *P* = 0.367). **(F)** Computer-assisted footprint in wheel running test for a representative mouse in each group. Right: gait analysis showed wider stride width and shorter stride length in sh*Mef2c* mice. Statistics were calculated using repeated measures ANOVA (*N* = 7 mice/group for shControl and *N* = 6 mice/group for shMef2c). **(G)** Number of rearing in cylinder test for shControl and shMef2c mice. Statistics were calculated using repeated measures ANOVA (*N* = 7 mice/group for shControl and *N* = 6 mice/group for shMef2c) (3weeks, ****, *P* = 0.01; 6weeks, ***, *P* = 0.05). Error bars represent means ± SEM.

**Supplementary Fig. 20.** **Behavioral tests at 9-weeks after intrathecal *Mef2c*-KD injection in mice. (A)** Number of forelimbs clasping in tail suspension test. **(B)** Number of rearing in cylinder test. **(C)** Minimal holding impulse in inverted grid test. Statistics were calculated using Student's t-test (*N* = 7 mice/group for shControl and *N* = 6 mice/group for shMef2c) (forelimb, *P* = 0.134; rearing, *P* = 0.638; holding, *P* = 0.144). **(D)** Gait analysis in accelerated wheel test. Statistics were calculated using Student's t-test (*N* = 7 mice/group for shControl and *N* = 6 mice/group for shMef2c) (width, *P* = 0.939; left, *P* = 0.848; right, *P* = 0.095). Error bars represent means ± SEM.

**Supplementary Fig. 21.** **Open field test analysis results of intrathecal *Mef2c*-KD injected mice.** **(A)** Comparing distance moved, cumulative duration and velocity of shControl and shMef2c mice in open-field test 3-weeks post-injection. Statistics were calculated using Student's t-test (*N* = 7 mice/group for shControl and *N* = 6 mice/group for shMef2c) (Distance, *P* = 0.73; inner, *P* = 0.076; velocity, *P* = 0.729). Error bars represent means ± SEM. **(B)** Variable being measured in each case. Left: track visualization used to obtain the total distance traveled of mouse (red shows the higher velocity). Right: heatmap visualization.

**Supplementary Table 1. The 27 SNPs predicated as the candidate risk-SNPs and -genes for ALS.**

| **Gene** | **SNP** | **Location** | ***P*-value ^GWAS^** |
| --- | --- | --- | --- |
| CLCN3, NEK1,  C4orf27 | rs11722212 | chr4:170534428 | 6.79E-05 |
| MEF2C | rs700587 | chr5:88110084 | 2.82E-04 |
| MEF2C | rs304153 | chr5:88121188 | 3.65E-04 |
| MEF2C | rs304152 | chr5:88124123 | 3.75E-04 |
| MEF2C | rs304151 | chr5:88125853 | 3.19E-04 |
| NEK1 | rs7687276 | chr4:170319471 | 3.55E-05 |
| NEK1 | rs6827421 | chr4:170326359 | 1.82E-05 |
| NEK1 | rs6831487 | chr4:170331972 | 1.46E-05 |
| MSRA | rs11989640 | chr8:10256054 | 3.59E-04 |
| TENM2 | rs4242226 | chr5:167181730 | 2.90E-05 |
| FOXP1 | rs9827299 | chr3:71353372 | 4.84E-04 |
| FOXP1 | rs76753627 | chr3:71356015 | 4.91E-04 |
| SDCCAG8 | rs58560561 | chr1:243537729 | 2.54E-04 |
| RBFOX1 | rs11077038 | chr16:6460736 | 3.85E-04 |
| ZNHIT3 | rs4796224 | chr17:34842521 | 3.66E-05 |
| NFIB | rs7021539 | chr9:14282792 | 4.46E-04 |
| PTPRD | rs10756021 | chr9:10292307 | 2.32E-04 |
| ZNF184 | rs7744110 | chr6:27421304 | 6.49E-05 |
| ZNF184 | rs2235252 | chr6:27422493 | 6.66E-05 |
| ZNF184 | rs2235254 | chr6:27422794 | 6.55E-05 |
| ZNF184 | rs12199110 | chr6:27423568 | 6.37E-05 |
| ZNF184 | rs2092121 | chr6:27424184 | 6.66E-05 |
| ZNF184 | rs2092122 | chr6:27424311 | 6.66E-05 |
| ZNF184 | rs764460 | chr6:27424374 | 6.43E-05 |
| ZNF184 | rs764461 | chr6:27424443 | 6.60E-05 |
| ZNF184 | rs10484398 | chr6:27424960 | 6.40E-05 |
| ZNF184 | rs6456789 | chr6:27426498 | 6.61E-05 |

**Supplementary Table 2. Information of human tissues from normal subjects and ALS patients.**

| **Number** | **Case (Normal or ALS)** | **Familial or Sporadic ALS** | **Sex** | **Age** | **PMI** |
| --- | --- | --- | --- | --- | --- |
| 1 | Normal |  | M | 70 | <24 |
| 2 | Normal |  | M | 84 | <24 |
| 3 | Normal |  | M | 60 | <24 |
| 4 | Normal |  | M | 69 | <24 |
| 5 | Normal |  | M | 60 | <24 |
| 1 | ALS | Sporadic | M | 84 | 27.5 |
| 2 | ALS | Sporadic | M | 45 | 51.6 |
| 3 | ALS | Sporadic | M | 60 | 43.6 |
| 4 | ALS | Sporadic | M | 78 | 25.7 |
| 5 | ALS | Sporadic | M | 62 | 19.4 |

**Supplementary Table 3. PCR primers that were used for pGL4.14-duplex-MEF2C promoter cloning.**

| **Primer name** | **Sequence (5’→3’)** | **Tm (℃)** |
| --- | --- | --- |
| MT-MEF2C_pro500-F1_BglII | ccggccAGATCTtggaaagattgattcaccaaga | 58 |
| MT-MEF2C_pro350-R150_HindIII | CATATTAAGCTTcctcacccctccagtctc | 57 |
| MT-MEF2C_pro200-R300_HindIII | CATATTAAGCTTcctttttctctccgtctctctc | 62 |
| MT-MEF2C_pro1-R500_HindIII | CGCATTAAGCTTacagcgtttgaaacatcgcgta | 59 |
| WT-MEF2C_pro500-F1_BglII | ccggccAGATCTtggaaagattgattcaccaaga | 53 |
| WT-MEF2C_pro350-R150_HindIII | CATATTAAGCTTcctcacccctccagtctc | 57 |
| WT-MEF2C_pro200-R300_HindIII | CATATTAAGCTTcctttttctctccgtctctctc | 56 |
| WT-MEF2C_pro1-R500_HindIII | CGCATTAAGCTTacagcgtttgaaacatcgcgta | 59 |

**Supplementary Table 4. PCR primers for NGS library generation**

| **Primer name** | **Sequence (5’→3’)** |
| --- | --- |
| NGS-1st_Foward | AGCGTATCTGAGTCATCATCAGA |
| NGS-1st_Reverse | TGCAGCAACTGGGCTTATGA |
| NGS-2nd_Foward | ACACTCTTTCCCTACACGACGCTCTTCCGATCTAGAGTGAAACTGCCATGCCA |
| NGS-2nd_Reverse | GTGACTGGAGTTCAGACGTGTGCTCTTCCGATCTTGTAAGTCAATCAAAAAGATCTGCT |

**Supplementary Table 5. qPCR primers that were used for determining the relative levels of gene expression.**

| **Model** | **Target** | **Primers** |
| --- | --- | --- |
| Mouse | *Nd2* | Forward: CCTCCTGGCCATCGTACTCA-3’  Reverse: GAATGGGGCGAGGCCTAGTT |
| Mouse | *Nd4* | Forward: GCCTACTCCTCAGTTAGCCACA  Reverse: GATGATGTGAGGCCATGTGCGA |
| Human | *ND4* | Forward: ACACTTATCCCCACCTTGGCTATC  Reverse: TTAGGGAGTCATAAGTGGAGTCCG |
| Mouse | *Nd5* | Forward: GGCCCTACACCAGTTTCAG  Reverse: AGGGCTCCGAGGCAAAGTA |
| Mouse | *12S rRNA* | Forward: CTAGCCACACCCCCACGGGA  Reverse: CGTATGACCGCGGTGGCTGG |
| Mouse | *Tfam* | Forward: GGCAAAGGATGATTCGGCTC  Reverse: CACTTCGTCCAACTTCAGCC |
| Mouse | *Mef2c* | Forward: CCACCCCTTCGAGATACCCACAA  Reverse: GGGAGTGGAATTCGTTCCGGTGA |
| Human | *MEF2C* | Forward: GTATGGCAATCCCCGAAACT  Reverse: CATCGTATTCTTGCTGCCTGG |
| Mouse | *Tomm20* | Forward: CACAAGGTGACTACGAGAAGGG  Reverse: AATGGTCGGAAGCTTGGTCAG |
| Mouse | *Gapdh* | Forward: TTTCCTCGTCCCGTAGACAAAA  Reverse: CGTTGAATTTGCCGTGAGTGG |

**Supplementary Table 6. qPCR primers that were used for MEF2C ChIP-qPCR.**

| **Model** | **Target** | **Primers** | **Mitochondrial DNA position** |
| --- | --- | --- | --- |
| Mouse | *Nd2* | Forward: ACTAACAGGATTCTTACCAAAATGA  Reverse: CCTAATATTTTCCACCCTAGCTATC | 4681  4882 |
| Mouse | *Nd4* | Forward: TACATAATTATTACCACCCAACGC  Reverse: TTCTTCTAACTACCAGTCCAAAAC | 11391  11500 |
| Mouse | *Nd5* | Forward: AAATACTGCAGCCCTACAAGCAAT  Reverse: GCAGCTACAGGAAAATCAGCAC | 12233  12396 |
| Mouse | *12SrRNA* | Forward: CTAGCCACACCCCCACGGGA  Reverse: CGTATGACCGCGGTGGCTGG | 214  309 |
| Mouse | *16SrRNA* | Forward: CGGCAAACAAGAACCCCGCC  Reverse: GTCAGGATACCGCGGCCGTT | 1912  2001 |
| Mouse | *Rgs6* | Forward: TACATCTGCAACCCAGTCATAAGT  Reverse: GGAGCGCTTTGTGGTTGATACTAT |  |

**Supplementary Table 7. Primer design sequences for shRNA-Mef2c plasmids.**

| **Number** | **Primer Sequence** | **shRNA Sequence** |
| --- | --- | --- |
| **shRNA 1** | **Forward:** TTTACAATGTCTGAGTTTGTCCGGCTCCTTCCT GTCAGAGCCGGACAAACTCAGACATTGATTTTT  **Reverse:** CTAGAAAAATCAATGTCTGAGTTTGTCCGGCTC TGACAGGAAGGAGCCGGACAAACTCAGACATTGT | GAGCCGGACAAACTCAGACATTG |
| **shRNA 2** | **Forward:** TTTGGTCTATGTGTTACACCAGGAGACCTTCCT GTCAGTCTCCTGGTGTAACACATAGACATTTTT  **Reverse:** CTAGAAAAATGTCTATGTGTTACACCAGGAGAC TGACAGGAAGGTCTCCTGGTGTAACACATAGACC | GTCTCCTGGTGTAACACATAGAC |
